# Sitting time and risk of cancer incidence and cancer mortality in postmenopausal women: the Women’s Health Accelerometry Collaboration

**DOI:** 10.1007/s10552-025-02024-0

**Published:** 2025-07-02

**Authors:** Eric T. Hyde, Kelly R. Evenson, Annie Green Howard, Humberto Parada, Jr., Chongzhi Di, Michael J. LaMonte, John Bellettiere, Carmen C. Cuthbertson, I-Min Lee, Andrea Z. LaCroix

**Affiliations:** 1 Present Address: Herbert Wertheim School of Public Health and Human Longevity Science, University of California, San Diego, 9500 Gilman Drive, La Jolla, CA 92093 USA; 2 Division of Epidemiology and Biostatistics, School of Public Health, San Diego State University, San Diego, CA USA; 3 Department of Epidemiology, Gillings School of Global Public Health, University of North Carolina at Chapel Hill, Chapel Hill, NC USA; 4 Department of Biostatistics, Gillings School of Global Public Health, University of North Carolina at Chapel Hill, Chapel Hill, NC USA; 5 Carolina Population Center, University of North Carolina at Chapel Hill, Chapel Hill, NC USA; 6 Division of Public Health Sciences, Fred Hutchinson Cancer Center, Seattle, WA USA; 7 Department of Epidemiology and Environmental Health, School of Public Health and Health Professions, University at Buffalo—SUNY, Buffalo, NY USA; 8 Department of Health Education and Promotion, East Carolina University, Greenville, NC USA; 9 Division of Preventive Medicine, Department of Medicine, Brigham and Women’s Hospital and Harvard Medical School, Boston, MA USA; 10 Department of Epidemiology, Harvard T. H. Chan School of Public Health, Boston, MA USA

**Keywords:** Sitting, Machine learning, Accelerometer, Bouts, Women’s health

## Abstract

**Purpose:**

Few studies have explored whether accelerometer-measured sedentary behavior increases cancer risk. We examined the associations of accelerometer-measured daily sitting time and mean sitting bout duration classified by the Convolutional Neural Network Hip Accelerometer Posture (CHAP) machine-learned algorithm with incidence of any cancer, incidence of 13 physical activity-related cancers, and cancer mortality among postmenopausal women.

**Methods:**

We used data from 22,097 women (mean age = 73.3 years, standard deviation [SD] = 6.7) in the Women’s Health Accelerometry Collaboration, a consortium of two US-based cohort studies of postmenopausal women: the Women’s Health Study and the Women’s Health Initiative Objective Physical Activity and Cardiovascular Health Study. Women who completed hip-worn triaxial accelerometry for ≥ 4 of 7 consecutive days were included. Associations between sedentary behaviors and physician-adjudicated invasive cancer incidence and mortality were tested using Cox regression.

**Results:**

Women were followed on average 8.0 years to identify cancer cases (*n* = 1,861) and deaths (*n* = 601). Overall, mean sitting time was 567 (SD = 113) min/day and mean sitting bout duration was 12.8 (SD = 4) min/bout. In covariate-adjusted models, one-SD increment higher in sitting time was associated with a 6% increased risk of incident cancer (hazard ratio [HR] = 1.06, 95% CI: 1.01–1.11); associations were similar for bout duration (HR = 1.05, 95% CI: 1.00–1.10). Estimates were similar for the 13 physical activity-related cancers (sitting time: HR = 1.10, 95% CI: 1.04–1.17; bout duration: HR = 1.08, 95% CI: 1.02–1.14) and for cancer mortality (sitting time: 1.06, 95% CI: 0.98–1.16; bout duration: HR = 1.05, 95% CI: 0.97–1.13).

**Conclusion:**

Among postmenopausal women, sedentary behavior was associated with increased cancer risk, particularly for physical activity-related cancers and cancer mortality.

**Supplementary Information:**

The online version contains supplementary material available at 10.1007/s10552-025-02024-0.

## Introduction

In the United States (US), cancer is the second leading cause of death among women, with an estimated 294,220 cancer-related deaths predicted to occur in 2025 [1]. However, it is estimated that 30–40% of cancers are preventable through modifiable risk factors, including increased physical activity and reduced sedentary behavior [2]. While substantial evidence indicates that higher physical activity levels are associated with reduced risk of cancer and cancer mortality [3], less is known about the role of sedentary behavior. Sedentary behavior refers to any waking behavior characterized by low energy expenditure (≤ 1.5 metabolic equivalents) while sitting, reclining, or lying [4]. The 2018 United States Physical Activity Guidelines Advisory Committee found moderate evidence for a relationship between greater time spent in sedentary behavior and higher risk of endometrial, colon, and lung cancers, while evidence grades could not be assigned for other site-specific cancers [3]. These conclusions were based on studies of self-reported sedentary time, which have been shown to be inaccurate [5], subject to recall bias, and result in underestimating the magnitude of associations of sedentary behavior with specific health outcomes [6, 7]. Accordingly, prospective studies employing device-based measurement of sedentary behavior and associations with cancer incidence and mortality are needed [3, 8]. In addition to measuring total sedentary time, accelerometers allow for the examination of sedentary bout duration, which is independently associated with some health risks [9, 10], but the relationship with cancer incidence is less clear [11, 12], Furthermore, the recently developed Convolutional Neural Network Hip Accelerometer Posture (CHAP) algorithm is a novel machine learning approach that more accurately classifies sitting and postural transitions from accelerometer output compared to cutpoint-based methods that are used by most accelerometer studies of sedentary behavior [13, 14].

The primary aim of this study was to examine the prospective associations of CHAP-classified daily sitting time and mean sitting bout duration with incidence of any cancer, a composite of 13 cancers previously found to be inversely associated with physical activity (combined 13) [15, 16], and cancer mortality in the Women’s Healthy Accelerometry Collaboration (WHAC), a combined prospective cohort of 22,097 older US women who participated in either the Women’s Health Initiative (WHI) Objective Physical Activity and Cardiovascular Health (OPACH) Study or the Women’s Health Study (WHS). We additionally evaluated effect modification by age, race/ethnicity, body mass index (BMI), cancer history at baseline, and moderate-to-vigorous physical activity (MVPA).

## Materials and methods

### Study population

Accelerometry, covariate, and site-specific incident cancer and cancer mortality data from WHS and OPACH were harmonized to comprise the WHAC. Details of WHS and OPACH history, participant recruitment, and methodology, and the WHAC harmonization have been previously described [17]. Briefly, the WHS is a completed randomized trial (1992–2004) that tested aspirin, beta-carotene, and vitamin E for the prevention of cardiovascular disease and cancer among 39,876 US women aged ≥ 45 years, who were subsequently followed observationally [18–20]. From 2011 to 2014, 18,289 WHS women participated in an ancillary study that collected accelerometry data; 17,466 (96%) women returned devices with usable data [21]. From 1993 to 1998, the WHI study initially recruited women 50–79 years for either a clinical trial(s) or an observational study from 40 clinical sites throughout the US. After data collection was completed in 2005, women were subsequently followed observationally. The OPACH study is an ancillary to the WHI Long Life Study, a 2012–2014 WHI substudy of postmenopausal women focused on healthy aging [22], and is a prospective study of accelerometry and chronic disease outcomes, including cancer [23]. Among the 9,252 women consented to the WHI Long Life Study, 7,048 participated in OPACH; 6,489 (92%) women returned accelerometers with usable data. All study protocols were approved by Institutional Review Boards of each participating institution and all women provided informed consent prior to participating in the studies.

### Accelerometer measures

Both the WHS and OPACH asked women to wear the ActiGraph GT3X + triaxial accelerometer (ActiGraph LLC, Pensacola, FL) for up to seven consecutive days [17]. In WHS, women were asked to wear the accelerometer over the right hip, removing it only during sleep or when in water [21]. In OPACH, women were asked to wear the accelerometer over the right hip including during sleep but not when in water [23]; subsequently, the time spent sleeping was removed for all analyses. Accelerometer non-wear time was removed using the validated Choi algorithm [24, 25]. For both cohorts, accelerometer wear adherence was defined as wearing time of ≥ 10 h on ≥ 4 days of device wear, consistent with other protocols [26].

Daily sitting time (min/day) and mean sitting bout duration (min) were classified using the CHAP algorithm. The CHAP algorithm is a machine-learned model architecture that was developed among 709 older adults in the Adult Changes in Thought Study (mean [SD] age = 77 [6.5] years; 59% women) who concurrently wore a GT3X + over the right hip and a thigh‐worn activPAL micro3 [13]. CHAP is intended for application on hip-worn ActiGraph GT3X + data and has strong agreement with activPAL micro3 classification of minute-level sitting compared to non-sitting [13]. It has been shown to measure sitting time and sit-to-stand transitions more accurately than using cutpoint methods [13].

Daily MVPA was derived from the raw 30-Hz accelerometry data for analysis based on the Accelerometer Activity Index (AAI) [27]. Average intensity per day was summarized as average AAI/15-s. OPACH calibration-study derived accelerometry cutpoints were applied to classify time in MVPA (≥ 587 AAI/15-s; min/day) [28, 29]. Accelerometer awake wear time was accounted for using the residuals method [30].

### Cancer outcomes

In WHS and OPACH, participants received annual mailed questionnaires in which they were asked to self-report new cancer diagnoses. Medical records were requested for all women who self-reported a diagnosis of cancer except those diagnosed with non-melanoma skin cancers [31]. Adjudicators reviewed the medical records; the date of cancer diagnosis was based on the earliest date of the relevant evidence (e.g., date of histologic confirmation). For cancers diagnosed only on death certificates without prior medical records, the date of death was used. Coding of cancer type was based on the International Classification of Diseases for Oncology (ICD-O-3) [32]. For both cohorts, deaths were reported by family members or postal authorities, with medical records, interviews with next of kin, and death certificates obtained to confirm the event. The National Death Index was also searched periodically.

The primary endpoints of interest for this study were: (1) incident invasive cancer at any site except non-melanoma skin cancer, (2) invasive combined 13 (bladder, breast, colon, endometrial, esophageal adenocarcinoma, gastric cardia, head and neck, kidney, liver, lung, myeloid leukemia, myeloma, and rectal cancer) previously found to be associated with low leisure-time physical activity [15], and (3) cancer mortality defined as death from any type of cancer. The most common site-specific cancers (breast, colon, endometrial, lung) were also assessed separately. Time-to-event was computed from the first day of accelerometry to the date of cancer event diagnosis through December 31, 2022 for WHS or February 19, 2022 for OPACH, with participants right-censored due to death from non-cancer causes or their last returned annual questionnaire.

### Covariates

Covariates were selected based on (1) their known or hypothesized associations with sedentary behavior and cancer and (2) whether they could be harmonized across both cohorts. Age, race/ethnicity (White, Black or African American, Hispanic or Latina, other or unknown), and education level (high school/GED or less, some college, college or more) were self-reported at enrollment into the original studies. Data on health history and health behaviors were ascertained annually, and data from the measure closest in time to accelerometer wear were used. Self-rated health was assessed with the question, ‘In general, would you say your health is excellent, very good, good, fair or poor?’ Women also reported on smoking status (current, former, never), alcohol intake (never, rarely, monthly, weekly, daily), current use of hormone therapy (yes/no), and history of cancer (yes/no), diabetes (yes/no), and cardiovascular disease (yes/no). Height and weight were self-reported in WHS and measured by study personnel in OPACH. BMI was calculated as weight (kg) divided by squared height (m^2^) and categorized as underweight (< 18.5), healthy weight (18.5–24.9), overweight (25.0–29.9), or obese (≥ 30.0 kg/m^2^) [33]. Physical function was based on responses to the RAND-36 (scores range from 0 to 100; higher scores reflect better function) [34].

### Statistical analysis

Sitting time and sitting bout duration were examined continuously and categorically by quartiles. Multivariable stratified Cox proportional hazards models [35] estimated hazard ratios (HRs) and 95% confidence intervals (CIs) for each cancer outcome in association with sitting time and bout duration. Stratified proportional hazards models were used with WHAC data to allow for baseline hazards of the two cohorts to differ [35]. Additionally, all models were a priori stratified by cohort. The proportional hazards assumption was inspected using plots of the Schoenfeld residuals and no violations were evident. To select a functional form of sitting time and bout duration, we conducted likelihood ratio tests (LRTs; *α* = 0.05) comparing a linear model to restricted cubic and quadratic models. Due to all LRTs having a *p* value > 0.05, the linear functional forms were used. We present results adjusted for age and race/ethnicity (Model 1) and a covariate-adjusted model (Model 2) which included adjustment for age, race/ethnicity, education, self-rated health, smoking, alcohol use, postmenopausal hormone therapy use, history of diabetes, and history of cardiovascular disease. The influence of physical activity on the association between sedentary behavior and cancer was evaluated by further adjusting Model 2 for MVPA. We also evaluated the impact of further adjusting Model 2 for BMI or physical function, as both are presumed confounders or mediators of the association between sedentary behavior and cancer.

Multiplicative interactions were evaluated using LRTs comparing Model 2 with cross-product terms for continuous sedentary variables with the reduced model without the interaction terms. Strata of interest explored for effect measure modification included age dichotomized at the sample median (< 75 vs. ≥ 75 years), race/ethnicity (White, Black or African American, and Hispanic or Latina), BMI (< 30 vs. ≥ 30 kg/m^2^), cancer history at baseline (yes vs. no), and MVPA dichotomized at the sample median (< 54 vs. ≥ 54 min/day). Using the 10th percentile as the referent group, HRs and 95% CIs were estimated for women in the 25th, 50th, 75th, and 90th percentiles of sedentary time to identify differences by stratification variables at extreme values. For sensitivity analyses, we repeated the analyses for cancer mortality but excluded women who died within the first 2 years of follow-up to evaluate the potential of confounding by health status. All analyses were conducted in R v4.0.2 (R Foundation for Statistical Computing; Vienna, Austria).

From the 23,955 WHAC participants with accelerometer data (*n* = 17,466 WHS and *n* = 6489 OPACH), we excluded 575 women (*n* = 319 WHS and *n* = 256 OPACH) with nonadherent accelerometer wear, 736 women with data on which the CHAP algorithm could not be successfully applied (*n* = 404 WHS and *n* = 332 OPACH), 404 women with missing covariate data (*n* = 283 WHS and *n* = 121 OPACH), 127 women who died within first year of accelerometry measurement (*n* = 56 WHS and *n* = 71 OPACH), and 16 WHS women who reported prevalent cancer post-trial randomization. The resulting analytic sample included 22,097 women (*n* = 16,388 WHS and *n* = 5,709 OPACH). For analyses of combined 13, 2000 women with any of the 13 cancers prevalent at baseline were excluded. For analyses of site-specific cancers, exclusions included women with prevalent breast cancer (*n* = 1340), colon cancer (*n* = 178), endometrial cancer or a hysterectomy (*n* = 9327), or lung cancer (*n* = 93) for each cancer outcome, respectively. Lastly, 1391 women were missing physical function data and were excluded from models adjusting for it.

## Results

### Description of cohort

**Fig. 1:**
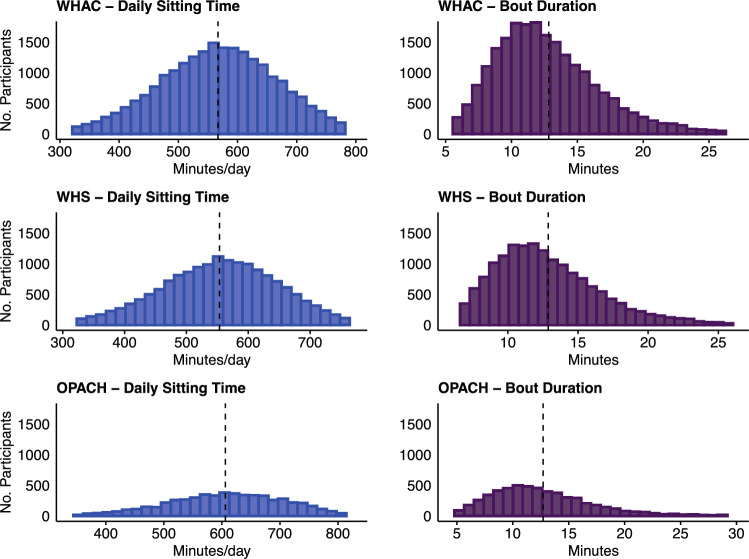
Distributions of CHAP-classified daily sitting time and mean sitting bout duration in WHAC, WHS, and OPACH. Abbreviations: CHAP, convolutional neural network hip accelerometer posture algorithm; OPACH, Objective Physical Activity and Cardiovascular Health Study; SD, standard deviation; WHAC, Women’s Health Accelerometry Collaboration; WHS, Women’s Health Study. The blue bars represent the distribution of daily sitting time (minutes/day) and the purple bars represent the distribution of mean sitting bout duration (min/bout). The dashed black lines represent the arithmetic mean of each distribution

Over a mean follow-up time of 8.0 years (range 0.1–11.6 years; 179,835 person-years), there were 1,861 incident cancers, of which 1,154 comprised the combined 13, 601 were cancer mortality cases, 632 were breast, 116 were colon, 128 were endometrial, and 207 were lung. The median (range) age at cancer diagnosis was 76.9 (63.3–98.7) years for cancer at any site, 76.1 (63.5–98.2) years for combined 13, 82.6 (65.1–98.7) years for cancer mortality, 75.2 (63.5–98.2) years for breast cancer, 81.9 (67.6–95.7) years for colon cancer, 74.4 (66.1–93.0) years for endometrial cancer, and 79.0 (65.7–94.3) years for lung cancer. Participant baseline characteristics stratified by quartiles of sitting time are presented in Table 1. Overall, participants were on average aged 73.3 (SD = 6.7) years, and majority were identified as non-Hispanic White (83.5%). Women in the highest sitting quartile versus lowest were older and more likely to have cancer, CVD, or diabetes and more likely to have higher BMI, lower frequency of alcohol use, worse self-rated general health, and wore physical function. The average number of adherent wear days was 6.7 (SD = 0.6) days [mean = 6.7, SD = 0.6 in WHS; mean = 6.5, SD = 0.7 in OPACH]. Average sitting time was 567 (SD = 113) min/day [mean = 553, SD = 107 in WHS; mean = 607, SD = 120 in OPACH]. For bout duration, the average was 12.8 (SD = 4) min and was the same in both cohorts. Distributions of sedentary behaviors overall and by cohort are displayed in Fig. 1. The Pearson correlation coefficient for sitting time and bout duration was 0.56.

**Table 1:** WHAC participant baseline characteristics overall and by quartiles of average daily sitting time

Characteristic	Total	Quartiles of sitting time (min/day)			
		< 493	493–566	567–639	> 639
*N*	22,097	5526	5523	5524	5524
Cohort, *n* (%)					
WHS	16,388 (74.2)	4632 (83.8)	4434 (80.3)	4071 (73.7)	3251 (58.9)
OPACH	5709 (25.8)	893 (16.2)	1090 (19.7)	1453 (26.3)	2273 (41.1)
Age (years), *n* (%)					
62–70	7830 (35.4)	2346 (42.5)	2101 (38.0)	1896 (34.3)	1487 (26.9)
70–79	9724 (44.0)	2462 (44.6)	2487 (45.0)	2460 (44.5)	2315 (41.9)
80–97	4543 (20.6)	717 (13.0)	936 (16.9)	1,168 (21.1)	1722 (31.2)
Mean [SD]	73.3 [6.7]	71.9 [6.0]	72.7 [6.3]	73.5 [6.7]	75.3 [7.3]
Race and ethnicity, *n *(%)					
Non-Hispanic White	18,460 (83.5)	4748 (85.9)	4746 (85.9)	4,558 (82.5)	4408 (79.8)
Non-Hispanic Black or African American	2,134 (9.7)	348 (6.3)	412 (7.5)	559 (10.1)	815 (14.8)
Hispanic or Latino	1,124 (5.1)	320 (5.8)	270 (4.9)	307 (5.6)	227 (4.1)
Other or unknown	379 (1.7)	109 (2.0)	96 (1.7)	100 (1.8)	74 (1.3)
Highest education level, *n* (%)					
High school/GED or less	1139 (5.2)	212 (3.8)	221 (4.0)	284 (5.1)	422 (7.6)
Some college	10,347 (46.8)	2756 (49.9)	2604 (47.1)	2541 (46.0)	2446 (44.3)
College graduate	10,611 (48.0)	2557 (46.3)	2699 (48.9)	2699 (48.9)	2656 (48.1)
Smoking status, *n* (%)					
Current	755 (3.4)	155 (2.8)	136 (2.5)	211 (3.8)	253 (4.6)
Former	9924 (44.9)	2470 (44.7)	2514 (45.5)	2451 (44.4)	2489 (45.1)
Never	11,418 (51.7)	2900 (52.5)	2874 (52.0)	2862 (51.8)	2782 (50.4)
Alcohol use, *n* (%)					
Never or rarely	8322 (37.7)	1966 (35.6)	1986 (36.0)	2076 (37.6)	2294 (41.5)
Monthly	3600 (16.3)	742 (13.4)	783 (14.2)	916 (16.6)	1159 (21.0)
Weekly	7244 (32.8)	1906 (34.5)	1920 (34.8)	1850 (33.5)	1568 (28.4)
Daily	2931 (13.3)	911 (16.5)	835 (15.1)	682 (12.4)	503 (9.1)
Body mass index (kg/m^2^), *n* (%)					
< 18.5	407 (1.84)	159 (2.9)	104 (1.9)	85 (1.54)	59 (1.1)
18.5–24.9	9,002 (40.7)	3035 (54.9)	2506 (45.4)	2021 (36.6)	1440 (26.1)
25.0–29.9	7,683 (34.8)	1,692 (30.6)	2003 (36.3)	2036 (36.9)	1952 (35.3)
≥ 30.0	3,365 (15.2)	475 (8.6)	682 (12.4)	973 (17.6)	1235 (22.4)
Mean [SD]	26.7 [5.3]	24.9 [4.3]	25.9 [4.5]	27.2 [5.1]	29.0 [6.0]
Self-rated general health, *n* (%)					
Excellent	4627 (20.94)	1441 (26.08)	1243 (22.5)	1150 (20.82)	793 (14.36)
Very good	10,553 (47.76)	2690 (48.69)	2787 (50.45)	2647 (47.92)	2429 (43.97)
Good	5968 (27.01)	1236 (22.37)	1309 (23.7)	1497 (27.1)	1926 (34.87)
Fair or poor	949 (4.29)	158 (2.86)	185 (3.35)	230 (4.16)	376 (6.81)
RAND-36 physical functioning score					
Mean [SD]	77.3 [23.0]	83.1 [19.4]	80.5 [21.1]	76.9 [22.6]	68.7 [25.9]
Missing (%)	1391 (6.3)	394 (7.1)	363 (6.6)	348 (6.3)	286 (5.2)
Daily MVPA (min/day)					
Mean [SD]	58.8 [34.0]	82.0 [38.5]	65.9 [29.4]	53.1 [25.0]	34.4 [20.9]
Current hormone therapy use, *n* (%)	1777 (8.0)	575 (10.4)	485 (8.8)	410 (7.4)	307 (5.6)
History of cardiovascular disease, *n* (%)	1254 (5.7)	216 (3.9)	268 (4.9)	324 (5.9)	446 (8.1)
History of diabetes, *n* (%)	2623 (11.9)	415 (7.5)	537 (9.7)	670 (12.1)	1,001 (18.1)
History of cancer, *n *(%)	2588 (11.7)	545 (9.9)	655 (11.9)	694 (13)	694 (12.6)

Abbreviations: GED, General Educational Development; SD, standard deviation; WHAC, Women’s Health Accelerometry Collaboration; OPACH, Objective Physical Activity and Cardiovascular Health Study; MVPA, moderate-to-vigorous physical activity; WHS, Women’s Health Study

### Associations of sitting time and bout duration with cancer

Results for incident cancer are shown in Table 2**.** In the covariate-adjusted model (Model 2), one-SD higher sitting time was associated with a 6% increased risk of incident cancer (HR = 1.06, 95% CI: 1.01–1.11) and a similar increased risk per one-SD longer bout duration (HR = 1.05, 95% CI: 1.00–1.10). HR point estimates were similar for both cohorts, although sitting time was statistically significant in OPACH only and bout duration was not statistically significant in either cohort, likely due to reduced sample sizes. Further adjustment for MVPA minimally affected point estimates, while adjusting for BMI or physical function attenuated all statistically significant estimates to the null.

**Table 2:** Associations of daily sitting time and mean sedentary bout duration with incident invasive cancer (*n* = 22,097)

	Continuous^b^	Quartiles of sedentary behavior^a^			
		Q1 (Low)	Q2	Q3	Q4 (High)
	HR (95% CI)	Ref	HR (95% CI)	HR (95% CI)	HR (95% CI)
WHAC					
Daily sitting time (min/day)					
Cancer cases [rate]^c^	1,861 [10.3]	434 [9.4]	423 [9.4]	482 [11.0]	522 [12.7]
Model 1^d^	1.07 (1.02, 1.12)	1 (ref)	0.96 (0.84, 1.10)	1.09 (0.96, 1.24)	1.16 (1.02, 1.32)
Model 2^d^	1.06 (1.01, 1.11)	1 (ref)	0.96 (0.84, 1.09)	1.07 (0.94, 1.23)	1.13 (0.98, 1.29)
Model 2 + MVPA^d^	1.07 (1.01, 1.13)	1 (ref)	0.97 (0.84, 1.11)	1.10 (0.95, 1.26)	1.16 (1.00, 1.35)
Model 2 + BMI^d^	1.03 (0.98, 1.09)	1 (ref)	0.94 (0.82, 1.07)	1.03 (0.90, 1.18)	1.05 (0.92, 1.21)
Model 2 + physical function^d^	1.04 (0.99, 1.10)	1 (ref)	0.96 (0.84, 1.10)	1.08 (0.94, 1.24)	1.09 (0.94, 1.25)
Bout duration (min)					
Cancer cases [rate]^c^	1,861 [10.3]	466 [10.4]	448 [9.9]	461 [10.4]	486 [11.5]
Model 1^d^	1.05 (1.01, 1.10)	1 (ref)	0.98 (0.86, 1.12)	1.02 (0.89, 1.16)	1.09 (0.96, 1.24)
Model 2^d^	1.05 (1.00, 1.10)	1 (ref)	0.98 (0.86, 1.12)	1.01 (0.89, 1.15)	1.08 (0.95, 1.23)
Model 2 + MVPA^d^	1.06 (1.01, 1.11)	1 (ref)	0.99 (0.86, 1.12)	1.02 (0.89, 1.17)	1.09 (0.95, 1.26)
Model 2 + BMI^d^	1.03 (0.98, 1.08)	1 (ref)	0.97 (0.85, 1.10)	0.98 (0.86, 1.12)	1.02 (0.89, 1.16)
Model 2 + physical function^d^	1.03 (0.99, 1.08)	1 (ref)	0.97 (0.85, 1.11)	1.00 (0.87, 1.14)	1.04 (0.91, 1.19)
WHS					
Daily sitting time (min/day)					
Cancer cases [rate]^c^	1,255 [9.3]	359 [9.1]	317 [8.7]	317 [9.5]	262 [10.2]
Model 1^d^	1.05 (0.99, 1.11)	1 (ref)	0.95 (0.82, 1.11)	1.04 (0.89, 1.21)	1.11 (0.94, 1.30)
Model 2^d^	1.04 (0.98, 1.10)	1 (ref)	0.94 (0.81, 1.10)	1.02 (0.88, 1.19)	1.09 (0.92, 1.28)
Model 2 + MVPA^d^	1.05 (0.98, 1.13)	1 (ref)	0.95 (0.81, 1.11)	1.04 (0.88, 1.22)	1.11 (0.93, 1.33)
Model 2 + BMI^d^	1.00 (0.93, 1.06)	1 (ref)	0.92 (0.79, 1.07)	0.97 (0.83, 1.13)	0.98 (0.82, 1.16)
Model 2 + physical function^d^	1.02 (0.95, 1.09)	1 (ref)	0.95 (0.81, 1.12)	1.02 (0.87, 1.20)	1.03 (0.86, 1.23)
Bout duration (min)					
Cancer cases [rate]^c^	1,255 [9.3]	295 [9.2]	324 [9.1]	314 [9.0]	322 [9.9]
Model 1^d^	1.04 (0.98, 1.10)	1 (ref)	0.98 (0.84, 1.15)	0.97 (0.83, 1.14)	1.06 (0.91, 1.24)
Model 2^d^	1.04 (0.98, 1.10)	1 (ref)	0.99 (0.84, 1.16)	0.97 (0.82, 1.14)	1.06 (0.90, 1.25)
Model 2 + MVPA^d^	1.05 (0.98, 1.12)	1 (ref)	0.99 (0.84, 1.16)	0.98 (0.82, 1.15)	1.08 (0.90, 1.29)
Model 2 + BMI^d^	1.00 (0.94, 1.07)	1 (ref)	0.97 (0.83, 1.13)	0.92 (0.79, 1.09)	0.97 (0.82, 1.15)
Model 2 + physical function^d^	1.02 (0.96, 1.09)	1 (ref)	0.99 (0.84, 1.17)	0.95 (0.80, 1.12)	1.03 (0.87, 1.22)
OPACH					
Daily sitting time (min/day)					
Cancer cases [rate]^c^	606 [13.5]	75 [11.4]	106 [12.3]	165 [15.6]	260 [16.6]
Model 1^d^	1.10 (1.02, 1.20)	1 (ref)	1.05 (0.78, 1.42)	1.30 (0.99, 1.71)	1.32 (1.02, 1.72)
Model 2^d^	1.08 (1.00, 1.18)	1 (ref)	1.07 (0.79, 1.43)	1.29 (0.98, 1.69)	1.28 (0.98, 1.66)
Model 2 + MVPA^d^	1.10 (1.01, 1.21)	1 (ref)	1.08 (0.80, 1.46)	1.32 (1.00, 1.75)	1.34 (1.01, 1.78)
Model 2 + BMI^d^	1.08 (0.99, 1.18)	1 (ref)	1.06 (0.79, 1.42)	1.27 (0.97, 1.68)	1.26 (0.96, 1.65)
Model 2 + physical function^d^	1.08 (0.99, 1.18)	1 (ref)	1.05 (0.78, 1.42)	1.29 (0.98, 1.71)	1.26 (0.96, 1.65)
Bout duration (min)					
Cancer cases [rate]^c^	606 [13.5]	171 [13.5]	124 [13.2]	147 [15.7]	164 [16.4]
Model 1^d^	1.07 (1.00, 1.14)	1 (ref)	0.96 (0.76, 1.21)	1.13 (0.91, 1.41)	1.13 (0.91, 1.41)
Model 2^d^	1.06 (0.99, 1.13)	1 (ref)	0.94 (0.75, 1.19)	1.10 (0.88, 1.38)	1.08 (0.87, 1.35)
Model 2 + MVPA^d^	1.06 (0.99, 1.14)	1 (ref)	0.95 (0.75, 1.20)	1.11 (0.88, 1.40)	1.10 (0.87, 1.39)
Model 2 + BMI^d^	1.05 (0.98, 1.13)	1 (ref)	0.94 (0.74, 1.18)	1.09 (0.87, 1.37)	1.06 (0.84, 1.33)
Model 2 + physical function^d^	1.05 (0.98, 1.13)	1 (ref)	0.93 (0.74, 1.18)	1.10 (0.88, 1.37)	1.05 (0.84, 1.32)

Abbreviations: BMI, body mass index; CHAP, convolutional neural network hip accelerometer posture algorithm; CI, confidence interval; HR, hazard ratio; MVPA, moderate-to-vigorous physical activity; OPACH, Objective Physical Activity and Cardiovascular Health Study; SD, standard deviation; WHAC, Women’s Health Accelerometry Collaboration; WHS, Women’s Health Study

^a^Quartile cut points for sitting time (min/day): < 493, 493–567, 568–639, and  > 639. For bout duration (min): < 9.9, 9.9–12.1, 12.2–15.0, and  > 15.0

^b^HR and 95% CI per one-SD increase in sedentary behavior. For sitting time, SDs were 113 min/day (WHAC), 107 min/day (WHS), and 120 min/day (OPACH). For bout duration, SDs were 4 min (WHAC and WHS) and 5 min (OPACH)

^c^Crude incidence rate per 1,000 person-years

^d^Model 1 is adjusted for age (years). Model 2 is adjusted for age (years), race and ethnicity (non-Hispanic White, non-Hispanic Black, Hispanic, other or unknown), education (high school/GED or less, some college, college graduate), smoking status (current, former, never), alcohol use (never or rarely, monthly, weekly, daily), general health (excellent or very good, good, fair, or poor), postmenopausal hormone use (current or otherwise), history of diabetes (yes or no), and history of cardiovascular disease (yes or no). Model 2 + MVPA is adjusted for Model 2 and daily MVPA (min/day). Model 2 + BMI is adjusted for Model 2 and body mass index (< 18.5, 18.5–24.9, 25.0–29.9, ≥ 30 kg/m^2^). Model 2 + physical function is adjusted for Model 2 and physical function (RAND-36 score)

Results for combined 13 cancers is shown in Table 3. In Model 2, one-SD higher sitting time was associated with a 10% increased risk of combined 13 (HR = 1.10, 95% CI:1.04–1.17) and an 8% increased risk for one-SD longer bout duration (HR = 1.08, 95% CI:1.02–1.14). Point estimates were similar by cohort, though statistically significant in WHS only. In WHAC, results were similar when adjusting for physical MVPA and physical function but slightly attenuated when adjusting for BMI.

**Table 3:** Associations of daily sitting time and mean sedentary bout duration with incidence of 13 cancers (*n* = 20,097)^a^

	Continuous^c^	Quartiles of Sedentary Behavior^b^			
		Q1 (Low)	Q2	Q3	Q4 (High)
	HR (95% CI)	Ref	HR (95% CI)	HR (95% CI)	HR (95% CI)
WHAC					
Daily sitting time (min/day)					
Cancer cases [rate]^d^	1,154 [6.9]	261 [6.1]	257 [6.1]	319 [7.9]	317 [8.4]
Model 1^e^	1.12 (1.05, 1.19)	1 (ref)	1.00 (0.84, 1.19)	1.27 (1.08, 1.50)	1.30 (1.09, 1.54)
Model 2^e^	1.10 (1.04, 1.17)	1 (ref)	0.99 (0.83, 1.18)	1.25 (1.06, 1.47)	1.25 (1.05, 1.49)
Model 2 + MVPA^e^	1.11 (1.03, 1.19)	1 (ref)	0.99 (0.83, 1.19)	1.26 (1.06, 1.49)	1.27 (1.05, 1.54)
Model 2 + BMI^e^	1.06 (0.99, 1.13)	1 (ref)	0.96 (0.81, 1.14)	1.18 (0.99, 1.39)	1.13 (0.95, 1.36)
Model 2 + physical function^e^	1.08 (1.02, 1.16)	1 (ref)	1.00 (0.84, 1.20)	1.24 (1.05, 1.47)	1.21 (1.01, 1.44)
Bout duration (min)					
Cancer cases [rate]^d^	1,154 [6.9]	276 [6.6]	281 [6.8]	285 [7.0]	312 [8.0]
Model 1^e^	1.08 (1.03, 1.15)	1 (ref)	1.04 (0.88, 1.23)	1.07 (0.90, 1.26)	1.21 (1.02, 1.42)
Model 2^e^	1.08 (1.02, 1.14)	1 (ref)	1.04 (0.88, 1.23)	1.06 (0.89, 1.25)	1.19 (1.01, 1.41)
Model 2 + MVPA^e^	1.08 (1.01, 1.15)	1 (ref)	1.04 (0.87, 1.23)	1.05 (0.88, 1.25)	1.18 (0.98, 1.41)
Model 2 + BMI^e^	1.05 (0.98, 1.11)	1 (ref)	1.02 (0.86, 1.20)	1.01 (0.85, 1.19)	1.09 (0.92, 1.29)
Model 2 + physical function^e^	1.07 (1.01, 1.13)	1 (ref)	1.03 (0.87, 1.22)	1.03 (0.87, 1.23)	1.15 (0.97, 1.37)
WHS					
Daily sitting time (min/day)					
Cancer cases [rate]^d^	831 [6.7]	218 [5.9]	205 [6.1]	226 [7.4]	182 [7.8]
Model 1^e^	1.12 (1.04, 1.20)	1 (ref)	1.03 (0.85, 1.24)	1.25 (1.03, 1.50)	1.29 (1.06, 1.57)
Model 2^e^	1.10 (1.02, 1.19)	1 (ref)	1.01 (0.84, 1.23)	1.22 (1.02, 1.48)	1.25 (1.02, 1.53)
Model 2 + MVPA^e^	1.12 (1.03, 1.22)	1 (ref)	1.03 (0.84, 1.25)	1.25 (1.03, 1.53)	1.29 (1.03, 1.62)
Model 2 + BMI^e^	1.04 (0.96, 1.12)	1 (ref)	0.98 (0.81, 1.19)	1.13 (0.93, 1.37)	1.08 (0.87, 1.34)
Model 2 + physical function^e^	1.08 (1.00, 1.17)	1 (ref)	1.03 (0.85, 1.26)	1.21 (0.99, 1.47)	1.20 (0.96, 1.48)
Bout duration (min)					
Cancer cases [rate]^d^	831 [6.7]	188 [6.3]	213 [6.5]	209 [6.5]	221 [7.4]
Model 1^e^	1.08 (1.01, 1.16)	1 (ref)	1.03 (0.84, 1.25)	1.03 (0.84, 1.25)	1.16 (0.95, 1.41)
Model 2^e^	1.07 (1.00, 1.15)	1 (ref)	1.03 (0.85, 1.25)	1.02 (0.84, 1.24)	1.15 (0.94, 1.40)
Model 2 + MVPA^e^	1.08 (1.00, 1.17)	1 (ref)	1.03 (0.84, 1.26)	1.02 (0.83, 1.26)	1.15 (0.92, 1.43)
Model 2 + BMI^e^	1.02 (0.95, 1.10)	1 (ref)	1.00 (0.82, 1.22)	0.95 (0.78, 1.16)	1.01 (0.83, 1.24)
Model 2 + physical function^e^	1.06 (0.99, 1.15)	1 (ref)	1.02 (0.83, 1.25)	0.99 (0.81, 1.21)	1.11 (0.90, 1.36)
OPACH					
Daily sitting time (min/day)					
Cancer cases [rate]^d^	323 [7.7]	43 [6.9]	52 [6.4]	93 [9.5]	135 [9.4]
Model 1^e^	1.11 (0.99, 1.24)	1 (ref)	0.90 (0.60, 1.36)	1.31 (0.91, 1.88)	1.26 (0.89, 1.80)
Model 2^e^	1.10 (0.98, 1.23)	1 (ref)	0.91 (0.60, 1.36)	1.30 (0.90, 1.87)	1.24 (0.87, 1.77)
Model 2 + MVPA^e^	1.08 (0.96, 1.23)	1 (ref)	0.90 (0.60, 1.35)	1.28 (0.88, 1.85)	1.20 (0.82, 1.75)
Model 2 + BMI^e^	1.09 (0.97, 1.22)	1 (ref)	0.89 (0.59, 1.34)	1.27 (0.88, 1.83)	1.20 (0.83, 1.72)
Model 2 + physical function^e^	1.09 (0.97, 1.22)	1 (ref)	0.90 (0.60, 1.35)	1.30 (0.90, 1.87)	1.20 (0.84, 1.72)
Bout duration (min)					
Cancer cases [rate]^d^	323 [7.7]	88 [7.4]	68 [7.8]	76 [8.6]	91 [10.0]
Model 1^e^	1.08 (0.99, 1.19)	1 (ref)	1.05 (0.76, 1.44)	1.16 (0.85, 1.58)	1.30 (0.96, 1.75)
Model 2^e^	1.08 (0.98, 1.19)	1 (ref)	1.05 (0.76, 1.44)	1.13 (0.83, 1.54)	1.28 (0.95, 1.73)
Model 2 + MVPA^e^	1.07 (0.97, 1.18)	1 (ref)	1.03 (0.75, 1.42)	1.11 (0.81, 1.52)	1.24 (0.90, 1.71)
Model 2 + BMI^e^	1.07 (0.97, 1.18)	1 (ref)	1.04 (0.75, 1.42)	1.11 (0.81, 1.52)	1.24 (0.91, 1.70)
Model 2 + physical function^e^	1.07 (0.97, 1.18)	1 (ref)	1.04 (0.75, 1.42)	1.12 (0.82, 1.53)	1.23 (0.90, 1.67)

Abbreviations: BMI, body mass index; CHAP, convolutional neural network hip accelerometer posture algorithm; CI, confidence interval; HR, hazard ratio; MVPA, moderate-to-vigorous physical activity; OPACH, Objective Physical Activity and Cardiovascular Health Study; SD, standard deviation; WHAC, Women’s Health Accelerometry Collaboration; WHS, Women’s Health Study

^a^The 13 cancers included bladder, breast, colon, endometrial, esophageal adenocarcinoma, gastric cardia, head and neck, kidney, liver, lung, myeloid leukemia, myeloma, and rectal cancer. 2,000 women were excluded from this analysis for having one of the 13 cancers prevalent at accelerometry baseline

^b^Quartile cut points for sitting time (min/day): < 493, 493–567, 568–639, and  > 639. For bout duration (min): < 9.9, 9.9–12.1, 12.2–15.0, and  > 15.0

^c^HR and 95% CI per one-SD increase in sedentary behavior. For sitting time, SDs were 113 min/day (WHAC), 107 min/day (WHS), and 120 min/day (OPACH). For bout duration, SDs were 4 min (WHAC and WHS) and 5 min (OPACH)

^d^Crude incidence rate per 1000 person-years

^e^Model 1 is adjusted for age (years). Model 2 is adjusted for age (years), race and ethnicity (non-Hispanic White, non-Hispanic Black, Hispanic, other or unknown), education (high school/GED or less, some college, college graduate), smoking status (current, former, never), alcohol use (never or rarely, monthly, weekly, daily), general health (excellent or very good, good, fair or poor), postmenopausal hormone use (current or otherwise), history of diabetes (yes or no), and history of cardiovascular disease (yes or no). Model 2 + MVPA is adjusted for Model 2 and daily MVPA (min/day). Model 2 + BMI is adjusted for Model 2 and body mass index (< 18.5, 18.5–24.9, 25.0–29.9, ≥ 30 kg/m^2^). Model 2 + physical function is adjusted for Model 2 and physical function (RAND-36 score)

Results for cancer mortality are shown in Table 4. Neither sitting time (HR per one-SD higher: 1.06, 95% CI: 0.98, 1.16) nor bout duration (HR per one-SD longer: 1.05, 95% CI: 0.97, 1.13) were significantly associated with cancer mortality. However, OPACH women had a 17% increased risk of cancer mortality (HR = 1.17, 95% CI: 1.04, 1.33) per one-SD higher sitting time and a 10% increased risk per one-SD longer bout duration (HR = 1.10, 95% CI: 1.00, 1.20). OPACH women in the highest (vs. lowest) quartile of sitting time had a 56% higher risk of cancer mortality (HR = 1.56, 95% CI: 1.05, 2.31). In all models, adjustment for BMI increased point estimates while adjustment for MVPA or physical function attenuated them.

**Table 4:** Associations of daily sitting time and mean sedentary bout duration with cancer mortality (N = 22,097)

	Continuous^b^	Quartiles of Sedentary Behavior^a^			
		Q1 (Low)	Q2	Q3	Q4 (High)
	HR (95% CI)	Ref	HR (95% CI)	HR (95% CI)	HR (95% CI)
WHAC					
Daily sitting time (min/day)					
Deaths [rate]^c^	601 [3.1]	115 [2.4]	139 [2.9]	130 [2.8]	217 [4.9]
Model 1^d^	1.12 (1.03, 1.22)	1 (ref)	1.10 (0.86, 1.41)	0.95 (0.74, 1.22)	1.34 (1.06, 1.69)
Model 2^d^	1.06 (0.98, 1.16)	1 (ref)	1.08 (0.84, 1.39)	0.89 (0.69, 1.15)	1.19 (0.93, 1.51)
Model 2 + MVPA^d^	1.02 (0.93, 1.13)	1 (ref)	1.05 (0.81, 1.34)	0.84 (0.65, 1.09)	1.08 (0.83, 1.40)
Model 2 + BMI^d^	1.09 (0.99, 1.19)	1 (ref)	1.10 (0.85, 1.41)	0.92 (0.72, 1.20)	1.26 (0.98, 1.62)
Model 2 + physical function^d^	1.05 (0.96, 1.16)	1 (ref)	1.03 (0.79, 1.35)	0.86 (0.65, 1.13)	1.15 (0.88, 1.49)
Bout duration (min)					
Deaths [rate]^c^	601 [3.1]	144 [3.0]	148 [3.1]	134 [2.9]	175 [3.9]
Model 1^d^	1.07 (1.00, 1.15)	1 (ref)	1.07 (0.85, 1.35)	0.95 (0.75, 1.21)	1.18 (0.95, 1.48)
Model 2^d^	1.05 (0.97, 1.13)	1 (ref)	1.06 (0.84, 1.34)	0.93 (0.73, 1.18)	1.12 (0.89, 1.40)
Model 2 + MVPA^d^	1.02 (0.94, 1.11)	1 (ref)	1.03 (0.82, 1.30)	0.88 (0.69, 1.12)	1.02 (0.80, 1.30)
Model 2 + BMI^d^	1.07 (0.99, 1.15)	1 (ref)	1.08 (0.85, 1.36)	0.96 (0.75, 1.21)	1.18 (0.93, 1.49)
Model 2 + physical function^d^	1.03 (0.95, 1.11)	1 (ref)	1.05 (0.82, 1.34)	0.93 (0.72, 1.20)	1.05 (0.82, 1.34)
WHS					
Daily sitting time (min/day)					
Deaths [rate]^c^	307 [2.1]	83 [2.0]	87 [2.3]	70 [2.0]	67 [2.5]
Model 1^d^	1.03 (0.92, 1.17)	1 (ref)	1.07 (0.79, 1.45)	0.92 (0.66, 1.26)	1.11 (0.80, 1.53)
Model 2^d^	0.95 (0.84, 1.08)	1 (ref)	1.03 (0.76, 1.39)	0.83 (0.60, 1.15)	0.91 (0.65, 1.27)
Model 2 + MVPA^d^	0.90 (0.79, 1.03)	1 (ref)	0.98 (0.72, 1.33)	0.76 (0.55, 1.07)	0.79 (0.55, 1.14)
Model 2 + BMI^d^	0.98 (0.86, 1.12)	1 (ref)	1.04 (0.77, 1.40)	0.86 (0.62, 1.19)	0.97 (0.69, 1.38)
Model 2 + physical function^d^	0.93 (0.81, 1.07)	1 (ref)	0.95 (0.68, 1.35)	0.77 (0.53, 1.11)	0.85 (0.58, 1.24)
Bout duration (min)					
Deaths [rate]^c^	307 [2.1]	67 [2.0]	90 [2.4]	64 [1.7]	86 [2.5]
Model 1^d^	1.01 (0.89, 1.13)	1 (ref)	1.15 (0.84, 1.58)	0.8 (0.57, 1.13)	1.14 (0.82, 1.57)
Model 2^d^	0.97 (0.86, 1.10)	1 (ref)	1.15 (0.84, 1.58)	0.78 (0.55, 1.10)	1.06 (0.76, 1.46)
Model 2 + MVPA^d^	0.92 (0.81, 1.06)	1 (ref)	1.10 (0.80, 1.52)	0.72 (0.50, 1.03)	0.94 (0.66, 1.35)
Model 2 + BMI^d^	0.99 (0.88, 1.13)	1 (ref)	1.16 (0.84, 1.59)	0.80 (0.56, 1.13)	1.12 (0.80, 1.57)
Model 2 + physical function^d^	0.93 (0.81, 1.07)	1 (ref)	1.20 (0.84, 1.72)	0.77 (0.52, 1.15)	0.99 (0.68, 1.45)
OPACH					
Daily sitting time (min/day)					
Deaths [rate]^c^	294 [6.1]	32 [4.6]	52 [5.7]	60 [5.3]	150 [9.0]
Model 1^d^	1.21 (1.07, 1.37)	1 (ref)	1.20 (0.77, 1.87)	1.07 (0.69, 1.65)	1.66 (1.12, 2.45)
Model 2^d^	1.17 (1.04, 1.33)	1 (ref)	1.20 (0.77, 1.87)	1.04 (0.68, 1.61)	1.56 (1.05, 2.31)
Model 2 + MVPA^d^	1.15 (1.00, 1.31)	1 (ref)	1.19 (0.76, 1.85)	1.01 (0.65, 1.57)	1.48 (0.97, 2.24)
Model 2 + BMI^d^	1.20 (1.06, 1.36)	1 (ref)	1.23 (0.79, 1.92)	1.09 (0.71, 1.69)	1.66 (1.11, 2.48)
Model 2 + physical function^d^	1.16 (1.02, 1.31)	1 (ref)	1.17 (0.75, 1.83)	1.04 (0.67, 1.60)	1.50 (1.01, 2.24)
Bout duration (min)					
Deaths [rate]^c^	294 [6.1]	77 [5.7]	58 [5.8]	70 [7.0]	89 [8.3]
Model 1^d^	1.12 (1.02, 1.22)	1 (ref)	0.96 (0.68, 1.34)	1.15 (0.83, 1.59)	1.23 (0.90, 1.68)
Model 2^d^	1.10 (1.00, 1.20)	1 (ref)	0.94 (0.67, 1.32)	1.12 (0.81, 1.55)	1.17 (0.85, 1.60)
Model 2 + MVPA^d^	1.08 (0.98, 1.19)	1 (ref)	0.92 (0.65, 1.29)	1.07 (0.77, 1.49)	1.07 (0.77, 1.50)
Model 2 + BMI^d^	1.12 (1.02, 1.22)	1 (ref)	0.95 (0.68, 1.34)	1.15 (0.82, 1.59)	1.22 (0.88, 1.69)
Model 2 + physical function^d^	1.08 (0.98, 1.19)	1 (ref)	0.91 (0.64, 1.28)	1.09 (0.78, 1.51)	1.09 (0.79, 1.51)

Abbreviations: BMI, body mass index; CHAP, convolutional neural network hip accelerometer posture algorithm; CI, confidence interval; HR, hazard ratio; MVPA, moderate-to-vigorous physical activity; OPACH, Objective Physical Activity and Cardiovascular Health Study; SD, standard deviation; WHAC, Women’s Health Accelerometry Collaboration; WHS, Women’s Health Study

^a^Quartile cut points for sitting time (min/day): < 493, 493–567, 568–639, and  > 639. For bout duration (min): < 9.9, 9.9–12.1, 12.2–15.0, and  > 15.0

^b^HR and 95% CI per one-SD increase in sedentary behavior. For sitting time, SDs were 113 min/day (WHAC), 107 min/day (WHS), and 120 min/day (OPACH). For bout duration, SDs were 4 min (WHAC and WHS) and 5 min (OPACH)

^c^Crude mortality rate per 1,000 person-years

^d^Model 1 is adjusted for age (years). Model 2 is adjusted for age (years), race and ethnicity (non-Hispanic White, non-Hispanic Black, Hispanic, other or unknown), education (high school/GED or less, some college, college graduate), smoking status (current, former, never), alcohol use (never or rarely, monthly, weekly, daily), general health (excellent or very good, good, fair or poor), postmenopausal hormone use (current or otherwise), history of diabetes (yes or no), and history of cardiovascular disease (yes or no). Model 2 + MVPA is adjusted for Model 2 and daily MVPA (min/day). Model 2 + BMI is adjusted for Model 2 and body mass index (< 18.5, 18.5–24.9, 25.0–29.9, ≥ 30 kg/m^2^). Model 2 + physical function is adjusted for Model 2 and physical function (RAND-36 score)

Our examination of effect measure modification of sitting time by age, race/ethnicity, BMI, cancer history, and MVPA is shown in Supplementary Table 1. Overall, there was no significant modification by any of the stratification variables (all *p *values > 0.05) indicating that associations for each outcome were generally similar across selected characteristics. Findings were similar for bout duration except for cancer history (Supplementary Table 2), which significantly modified the association between sitting bout duration and cancer mortality (p-interaction = 0.049). Estimates were higher among those without cancer history (HR per one-SD higher: 1.08, 95% CI: 1.02, 1.15) than those with cancer history (HR = 0.80, 95% CI: 0.56, 1.13). Results from our analyses of site-specific cancers are shown in Supplementary Tables 3–6. Higher sitting time and longer bout duration were associated with increases in risk of breast, endometrial, and lung cancers; however, 95% CIs included the null. Associations for colon cancer were generally null, but analyses were limited by low numbers of cases. Sensitivity analyses for cancer mortality that excluded women who died during the first 2 years of follow-up were consistent in direction and magnitude with the primary results (Supplementary Table 7).

## Discussion

Among 22,097 postmenopausal ambulatory women, higher amounts of CHAP-classified daily sitting time and longer bout duration were associated with increased risk of any cancer and a composite of 13 physical activity-related cancers even after adjusting for daily MVPA, while associations with cancer mortality were not statistically significant. Additional adjustment for either BMI or physical function attenuated most associations except for cancer mortality, where adjusting for BMI increased point estimates. Effect modification analyses indicated associations did not significantly differ across selected subgroups. Our findings are generally consistent with studies reporting an association of higher self-reported sedentary behavior with an increased risk of some cancers [3, 8, 36] and build upon two previous WHI studies of self-reported sitting time with breast [37] and lung [38] cancer incidence. Among WHI women, Wang et al. reported that more time spent sitting was associated with a 16% increased risk for lung cancer (≥ 10 vs. ≤ 5 h/day) [38], while Nomura et al. reported a null association with breast cancer incidence (≥ 10 vs. ≤ 5 h/day) [37]. For comparison, our study reported associations between accelerometer-measured sitting time and lung cancer (≥ 10.7 vs. ≤ 8.2 h/day HR: 1.18, 95% CI: 0.77–1.80) and breast cancer (≥ 10.7 vs. ≤ 8.2 h/day HR: 1.10, 95% CI: 0.87–1.40). The different associations found in our study may be due in part to the use of accelerometry, which is more accurate and less prone to bias than self-reported sitting time [6], and the older age distribution of women at accelerometer measurement.

Few other studies have assessed the relationships between accelerometer-measured sedentary behavior and cancer outcomes, and the results have been mixed [11, 12, 39–42]. For cancer incidence, two separate studies of EPIC-Norfolk participant data reported a linear association for both daily sitting time [39] and bout duration [12], while a Swedish study of sitting time was not significant but estimates were in the harmful direction [40]. For breast cancer, a 2022 Mendelian randomization study of UK Biobank participants found greater accelerometer-measured sedentary time was associated with higher risk for hormone receptor-negative types only [41], and a case–control study of Polish women found sedentary time was positively associated with breast cancer odds [42]. A prospective study of cancer mortality from the REGARDS cohort reported a significant association with higher sitting time but a non-significant association for bout duration [11]. Some reasons for why our studies results differ from previous studies include the differences in age (e.g., mean age in UK Biobank was 61 years compared to 73 years in WHAC) in addition to differences in accelerometer wear location, device brand, and data processing methods used. In addition, the imprecision of cutpoint methods used for determining sedentary behavior in previous studies may have a role. This method is based on movement and does not account for posture, an important aspect of sedentary behavior [4, 43]. Our study strengthens the evidence base using the CHAP algorithm, which is superior to cutpoint methods for measuring both sitting time and posture [13, 44]. Our study is one of the first to examine accelerometer-measured sedentary behavior in relation to cancer in older postmenopausal women exclusively and provides a comprehensive analysis of the association across multiple cancer outcomes.

The precise mechanisms linking sedentary behavior to cancer incidence are unclear though several have been hypothesized. Adiposity accumulated through sedentary behavior is likely both an independent and a mediating variable on pathways with cancer risk [45], which is why our analysis adjusted for BMI separately in fully adjusted models. Prolonged sitting is also associated with increased chronic inflammation and production of pro-inflammatory cytokines, which can promote cellular damage and mutagenesis [2]. Dysregulation of metabolic pathways and alterations in sex hormones can result from higher levels of sedentary behavior, which are also associated with increased cancer risk [2, 45]. While other hypotheses are still emerging, a better understanding these biologic mechanisms can help inform targeted interventions to mitigate the impact of sedentary behavior on cancer risk.

For an approximate 2-h/day increment in higher sitting time at baseline, we observed a 6% increased risk of overall cancer incidence and a 10% increased risk of the combined 13. These associations are similar in magnitude (though in opposite direction) with those from studies of physical activity and cancer risk [16, 46]. Furthermore, the associations remained significant after adjusting for MVPA, which suggests that older women could lower their cancer risk by reducing sitting time regardless of their physical activity levels. Reducing sedentariness may be a more feasible behavioral modification than increasing MVPA, and recent randomized controlled trials have demonstrated the effectiveness of interventions to reduce sitting time in older adults [47, 48]. Thus, a reduction in cancer risk of up to 10% could be meaningful at both the individual and population level. Given that older US women spend a substantial portion the day in sedentary behaviors [49], sitting less and for shorter bouts may help reduce cancer risk and is likely to benefit many other aspects of healthy aging including mobility and cardiovascular risk [47, 48].

### Strengths and limitations

Strengths of our study include the harmonization of accelerometry, covariate, and cancer data from WHS and OPACH to create a large sample of older US women with usable hip-worn accelerometry data. We utilized the CHAP algorithm to determine sedentary behaviors, which is more accurate that cutpoint-based methods [13, 44]. While women wore an accelerometer on the hip for up to 7 days under normal living conditions, the inability to measure changes in sedentary behaviors over time represents a limitation of the present study [50]. In addition, evaluating the combined 13 composite outcome complicates the attribution of specific biologic mechanisms that might explain the observed associations, because the site-specific cancers may involve both shared and unique mechanisms by which sedentary behavior influences risk. The harmonization of covariate data across cohorts resulted in the re-categorization of some variables, such as smoking status, which could result in residual confounding. We examined associations of sedentary behavior with some site-specific cancers, although our analyses of endometrial, lung, and colon cancers may have been underpowered due to low numbers of events. We were unable to control for some comorbidities that may be associated with both sedentary behavior and cancer, such as arthritis and chronic obstructive pulmonary disease, although diabetes, cardiovascular disease, and cancer histories were included. In addition, we were unable to control for other confounders, such as oral contraceptive use and parity that are associated with some site-specific cancers. Lastly, our findings warrant replication in cohorts that include male and younger participants to assess generalizability.

## Conclusion

In a sample of over 22,000 older US women, higher amounts of accelerometer-measured daily sitting time and longer bouts were associated with increased risk for cancer incidence and mortality. These findings add to a growing body of evidence indicating that limiting sedentary behaviors may help reduce risk of cancers. Additionally, our results support public health messaging encouraging older adults to sit less and move more throughout the day to promote healthy longevity.

## Supplementary Information

Below is the link to the electronic supplementary material.

Supplementary file1 (DOCX 106 kb)

## Data Availability

Data are available by following the data sharing policies of the Women’s Health Study and Women’s Health Initiative accessed at the following websites. Women’s Health Study data policies are available at https://whs.bwh.harvard.edu/ and the Women’s Health Initiative data sharing policies are available at https://www.whi.org/md/working-with-whi-data.

## Abbreviations

AAI: Accelerometer Activity Index
BMI: Body mass index
CHAP: Convolutional Neural Network Hip Accelerometer Posture
CI: Confidence interval
HR: Hazard ratio
IDC-O-3: International Classification of Diseases for Oncology
LRT: Likelihood ratio test
MVPA: Moderate-to-vigorous physical activity
OPACH: Objective Physical Activity and Cardiovascular Health
SD: Standard deviation
US: United States
WHAC: Women’s Healthy Accelerometry Collaboration
WHI: Women’s Health Initiative
WHS: Women’s Health Study
