# Supplementary material for: Sitting time and risk of cancer incidence and cancer mortality in postmenopausal women: the Women’s Health Accelerometry Collaboration
Source: Cancer Causes Control. 2025 Jul 2;36(11):1417–30. doi: 10.1007/s10552-025-02024-0 (PMC12578691; doi:10.1007/s10552-025-02024-0)
Supplement: Supplementary file 1 — Supplementary file1 (DOCX 106 kb) [file 10552_2025_2024_MOESM1_ESM.docx]

**Supplementary Online Content**

**Supplemental Table 1.** Associations of CHAP-classified daily sitting time with cancer outcomes among cohort subgroups in WHAC

**Supplemental Table 2.** Associations of CHAP-classified minutes of mean sitting bout duration with cancer outcomes among cohort subgroups in WHAC

**Supplemental Table 3.** Associations of CHAP-classified minutes of daily sitting time and mean sitting bout duration with breast cancer incidence (N=20,757)

**Supplemental Table 4.** Associations of CHAP-classified minutes of daily sitting time and mean sitting bout duration with endometrial cancer incidence (N=12,770)

**Supplemental Table 5.** Associations of CHAP-classified minutes of daily sitting time and mean sitting bout duration with lung cancer incidence (N=22,004)

**Supplemental Table 6.** Associations of CHAP-classified minutes of daily sitting time and mean sitting bout duration with colon cancer incidence (N=21,919)

**Supplemental Table 7.** Associations of CHAP-classified minutes of daily sitting time and mean sitting bout duration with fatal cancer after excluding deaths within the first 2 years of follow-up (n=70 deaths excluded)

**Supplemental Table 1.** Associations of CHAP-classified daily sitting time with cancer outcomes among cohort subgroups in WHAC

| **Subgroup by Outcome** | **Sample Size** | **Events** | **Percentiles of Sitting Time^a^** | | | | | **Continuous**  **(1-SD)** | ***P*-value^b^** |
| --- | --- | --- | --- | --- | --- | --- | --- | --- | --- |
|  |  |  | **10^th^ Percentile** | **25^th^ Percentile** | **50^th^ Percentile** | **75^th^ Percentile** | **90^th^ Percentile** |  |  |
|  | **n** | **n (%)** | **Ref** | **HR (95% CI)** | **HR (95% CI)** | **HR (95% CI)** | **HR (95% CI)** | **HR (95% CI)** |  |
| **Any Cancer** | | | | | | | | | |
| Overall | 22,097 | 1,861 (8.4) | 1.00 | 1.04 (1.01, 1.08) | 1.09 (1.02, 1.16) | 1.14 (1.03, 1.25) | 1.18 (1.05, 1.34) | 1.07 (1.02, 1.12) | - |
| Age (years) |  |  |  |  |  |  |  |  |  |
| <75 | 13,844 | 1,126 (8.1) | 1.00 | 1.04 (1.00, 1.09) | 1.09 (1.00, 1.18) | 1.14 (1.01, 1.29) | 1.18 (1.01, 1.39) | 1.07 (1.00, 1.15) | 0.44 |
| ≥75 | 8,253 | 735 (8.9) | 1.00 | 1.06 (1.01, 1.12) | 1.13 (1.03, 1.25) | 1.21 (1.04, 1.40) | 1.28 (1.05, 1.55) | 1.10 (1.02, 1.19) |  |
| Race and ethnicity |  |  |  |  |  |  |  |  |  |
| White | 18,460 | 1,523 (8.3) | 1.00 | 1.04 (1.01, 1.08) | 1.09 (1.02, 1.17) | 1.14 (1.02, 1.27) | 1.19 (1.03, 1.36) | 1.07 (1.01, 1.13) | 0.85 |
| Black or African American | 2,134 | 222 (10.4) | 1.00 | 1.05 (0.96, 1.14) | 1.11 (0.93, 1.32) | 1.16 (0.89, 1.51) | 1.22 (0.87, 1.71) | 1.08 (0.94, 1.23) |  |
| Hispanic or Latina | 1,124 | 93 (8.3) | 1.00 | 1.05 (0.91, 1.20) | 1.10 (0.82, 1.46) | 1.15 (0.74, 1.77) | 1.20 (0.68, 2.11) | 1.07 (0.86, 1.34) |  |
| BMI (kg/m^2^) |  |  |  |  |  |  |  |  |  |
| <30 | 17,092 | 1,380 (8.1) | 1.00 | 1.02 (0.98, 1.06) | 1.04 (0.97, 1.12) | 1.06 (0.95, 1.19) | 1.08 (0.93, 1.25) | 1.03 (0.97, 1.09) | 0.45 |
| ≥30 | 5,005 | 481 (9.6) | 1.00 | 1.07 (1.00, 1.14) | 1.15 (1.01, 1.31) | 1.23 (1.01, 1.51) | 1.31 (1.01, 1.70) | 1.11 (1.01, 1.23) |  |
| Cancer history |  |  |  |  |  |  |  |  |  |
| No | 19,509 | 1,653 (8.5) | 1.00 | 1.05 (1.01, 1.08) | 1.10 (1.03, 1.18) | 1.16 (1.05, 1.28) | 1.21 (1.06, 1.38) | 1.08 (1.02, 1.14) | 0.93 |
| Yes | 2,588 | 208 (8.0) | 1.00 | 0.99 (0.90, 1.09) | 0.98 (0.82, 1.18) | 0.98 (0.74, 1.29) | 0.97 (0.67, 1.39) | 0.99 (0.86, 1.14) |  |
| Daily MVPA (min/day) |  |  |  |  |  |  |  |  |  |
| <54 | 11,042 | 961 (8.7) | 1.00 | 1.07 (1.03, 1.12) | 1.16 (1.06, 1.27) | 1.25 (1.09, 1.43) | 1.33 (1.11, 1.60) | 1.12 (1.04, 1.20) | 0.11 |
| ≥54 | 11,055 | 900 (8.2) | 1.00 | 1.02 (0.97, 1.07) | 1.03 (0.93, 1.14) | 1.05 (0.90, 1.22) | 1.07 (0.88, 1.30) | 1.03 (0.95, 1.11) |  |
| **13 Physical Activity-Related Cancers** | | | | | | | | | |
| Overall | 20,097 | 1,154 (5.7) | 1.00 | 1.07 (1.03, 1.11) | 1.15 (1.06, 1.24) | 1.23 (1.09, 1.39) | 1.31 (1.12, 1.53) | 1.11 (1.04, 1.18) | - |
| Age (years) |  |  |  |  |  |  |  |  |  |
| <75 | 12,700 | 742 (5.8) | 1.00 | 1.09 (1.03, 1.14) | 1.18 (1.07, 1.31) | 1.29 (1.11, 1.50) | 1.39 (1.14, 1.70) | 1.14 (1.05, 1.23) | 0.35 |
| ≥75 | 7,397 | 412 (5.6) | 1.00 | 1.05 (0.99, 1.13) | 1.12 (0.98, 1.27) | 1.18 (0.97, 1.44) | 1.24 (0.96, 1.61) | 1.09 (0.98, 1.20) |  |
| Race and ethnicity |  |  |  |  |  |  |  |  |  |
| White | 16,743 | 963 (5.8) | 1.00 | 1.08 (1.03, 1.13) | 1.17 (1.07, 1.28) | 1.26 (1.10, 1.44) | 1.35 (1.13, 1.61) | 1.13 (1.05, 1.21) | 0.52 |
| Black or African American | 1,968 | 123 (6.3) | 1.00 | 1.05 (0.94, 1.18) | 1.12 (0.88, 1.42) | 1.18 (0.82, 1.69) | 1.24 (0.78, 1.97) | 1.09 (0.91, 1.31) |  |
| Hispanic or Latina | 1,041 | 51 (4.9) | 1.00 | 0.95 (0.78, 1.15) | 0.89 (0.60, 1.34) | 0.85 (0.46, 1.55) | 0.80 (0.37, 1.76) | 0.92 (0.67, 1.25) |  |
| BMI (kg/m^2^) |  |  |  |  |  |  |  |  |  |
| <30 | 15,553 | 844 (5.4) | 1.00 | 1.04 (0.99, 1.09) | 1.08 (0.98, 1.19) | 1.12 (0.97, 1.30) | 1.16 (0.96, 1.40) | 1.06 (0.98, 1.14) | 0.35 |
| ≥30 | 4,544 | 310 (6.8) | 1.00 | 1.10 (1.01, 1.20) | 1.22 (1.03, 1.44) | 1.35 (1.05, 1.74) | 1.47 (1.06, 2.05) | 1.16 (1.02, 1.33) |  |
| Cancer history |  |  |  |  |  |  |  |  |  |
| No | 19,509 | 1,106 (5.7) | 1.00 | 1.07 (1.03, 1.11) | 1.15 (1.05, 1.24) | 1.23 (1.08, 1.39) | 1.30 (1.11, 1.53) | 1.11 (1.04, 1.18) | 0.89 |
| Yes | 588 | 48 (8.2) | 1.00 | 1.05 (0.85, 1.28) | 1.10 (0.72, 1.67) | 1.15 (0.61, 2.17) | 1.20 (0.53, 2.73) | 1.07 (0.78, 1.48) |  |
| Daily MVPA (min/day) |  |  |  |  |  |  |  |  |  |
| <54 | 9,919 | 587 (5.9) | 1.00 | 1.08 (1.02, 1.15) | 1.18 (1.04, 1.33) | 1.28 (1.07, 1.53) | 1.37 (1.09, 1.73) | 1.13 (1.03, 1.24) | 0.50 |
| ≥54 | 10,178 | 567 (5.6) | 1.00 | 1.05 (0.98, 1.11) | 1.10 (0.97, 1.25) | 1.15 (0.95, 1.40) | 1.20 (0.94, 1.55) | 1.08 (0.98, 1.19) |  |
| **Fatal Cancer** | | | | | | | | | |
| Overall | 22,097 | 601 (2.7) | 1.00 | 1.05 (1.00, 1.11) | 1.12 (1.00, 1.25) | 1.18 (1.00, 1.39) | 1.24 (1.00, 1.54) | 1.09 (1.00, 1.18) | - |
| Age (years) |  |  |  |  |  |  |  |  |  |
| <75 | 13,844 | 241 (1.8) | 1.00 | 1.03 (0.95, 1.12) | 1.06 (0.89, 1.27) | 1.10 (0.84, 1.43) | 1.13 (0.80, 1.59) | 1.05 (0.92, 1.20) | 0.45 |
| ≥75 | 8,253 | 360 (4.4) | 1.00 | 1.12 (1.04, 1.20) | 1.25 (1.09, 1.45) | 1.41 (1.13, 1.75) | 1.56 (1.17, 2.07) | 1.19 (1.07, 1.33) |  |
| Race and ethnicity |  |  |  |  |  |  |  |  |  |
| White | 18,460 | 449 (2.4) | 1.00 | 1.04 (0.97, 1.11) | 1.08 (0.95, 1.23) | 1.12 (0.92, 1.37) | 1.17 (0.90, 1.50) | 1.06 (0.96, 1.17) | 0.38 |
| Black or African American | 2,134 | 99 (4.6) | 1.00 | 1.19 (1.04, 1.37) | 1.44 (1.08, 1.92) | 1.73 (1.13, 2.66) | 2.04 (1.17, 3.56) | 1.33 (1.06, 1.65) |  |
| Hispanic or Latina | 1,124 | 45 (4.0) | 1.00 | 0.95 (0.78, 1.15) | 0.89 (0.60, 1.33) | 0.84 (0.46, 1.54) | 0.80 (0.36, 1.75) | 0.92 (0.67, 1.25) |  |
| BMI (kg/m^2^) |  |  |  |  |  |  |  |  |  |
| <30 | 17,092 | 471 (2.8) | 1.00 | 1.05 (0.99, 1.12) | 1.10 (0.97, 1.26) | 1.16 (0.96, 1.41) | 1.21 (0.94, 1.56) | 1.08 (0.98, 1.19) | 0.07 |
| ≥30 | 5,005 | 130 (2.6) | 1.00 | 1.17 (1.03, 1.33) | 1.38 (1.06, 1.81) | 1.63 (1.09, 2.43) | 1.88 (1.12, 3.17) | 1.28 (1.05, 1.57) |  |
| Cancer history |  |  |  |  |  |  |  |  |  |
| No | 19,509 | 430 (2.2) | 1.00 | 1.02 (0.96, 1.09) | 1.05 (0.92, 1.20) | 1.08 (0.89, 1.31) | 1.10 (0.85, 1.42) | 1.04 (0.94, 1.15) | 0.21 |
| Yes | 2,588 | 171 (6.6) | 1.00 | 1.11 (1.00, 1.24) | 1.25 (1.00, 1.55) | 1.39 (1.00, 1.93) | 1.54 (1.01, 2.35) | 1.18 (1.00, 1.40) |  |
| Daily MVPA (min/day) |  |  |  |  |  |  |  |  |  |
| <54 | 11,042 | 403 (3.6) | 1.00 | 1.06 (0.99, 1.13) | 1.12 (0.98, 1.29) | 1.19 (0.97, 1.47) | 1.26 (0.96, 1.65) | 1.09 (0.98, 1.22) | 0.26 |
| ≥54 | 11,055 | 198 (1.8) | 1.00 | 0.98 (0.89, 1.09) | 0.96 (0.78, 1.19) | 0.95 (0.69, 1.31) | 0.93 (0.61, 1.42) | 0.97 (0.83, 1.15) |  |
| Abbreviations: BMI, body mass index; CHAP, convolutional neural network hip accelerometer posture algorithm; CI, confidence interval; HR, hazard ratio; MVPA, moderate-to-vigorous physical activity; SD, standard deviation; WHAC, Women’s Health Accelerometry Collaboration.  Models were adjusted for age, race and ethnicity, education, smoking status, alcohol use, self-rated general health, postmenopausal hormone use, history of diabetes, and history of cardiovascular disease. Models for each level of race and ethnicity were not mutually adjusted for race and ethnicity.  ^a^ Corresponding percentile values (min/day): 425.7 (10^th^), 492.8 (25^th^), 566.6 (50^th^), 639.5 (75^th^), 709.6 (90^th^).  ^b^ P-value for multiplicative interaction from likelihood ratio tests | | | | | | | | | |

| **Supplemental Table 2.** Associations of CHAP-classified minutes of mean sitting bout duration with cancer outcomes among cohort subgroups in WHAC | | | | | | | | | |
| --- | --- | --- | --- | --- | --- | --- | --- | --- | --- |
| **Subgroup by Outcome** | **Sample Size** | **Events** | **Percentiles of Mean Sitting Bout Duration^a^** | | | | | **Continuous**  **(1-SD)** | ***P*-value^b^** |
|  |  |  | **10^th^ Percentile** | **25^th^ Percentile** | **50^th^ Percentile** | **75^th^ Percentile** | **90^th^ Percentile** |  |  |
|  | **n** | **n (%)** | **Ref** | **HR (95% CI)** | **HR (95% CI)** | **HR (95% CI)** | **HR (95% CI)** | **HR (95% CI)** |  |
| **Any Cancer** | | | | | | | | | |
| Overall | 22,097 | 1,861 (8.4) | 1.00 | 1.02 (1.00, 1.03) | 1.04 (0.99, 1.08) | 1.06 (0.99, 1.14) | 1.09 (0.99, 1.21) | 1.04 (0.99, 1.09) | - |
| Age (years) |  |  |  |  |  |  |  |  |  |
| <75 | 13,844 | 1,126 (8.1) | 1.00 | 1.02 (1.00, 1.05) | 1.06 (1.00, 1.12) | 1.10 (1.00, 1.21) | 1.14 (0.99, 1.32) | 1.06 (1.00, 1.13) | 0.41 |
| ≥75 | 8,253 | 735 (8.9) | 1.00 | 1.01 (0.99, 1.05) | 1.03 (0.97, 1.08) | 1.05 (0.95, 1.16) | 1.07 (0.92, 1.25) | 1.03 (0.97, 1.10) |  |
| Race and ethnicity |  |  |  |  |  |  |  |  |  |
| White | 18,460 | 1,523 (8.3) | 1.00 | 1.02 (1.00, 1.04) | 1.04 (0.92, 1.11) | 1.07 (0.99, 1.16) | 1.11 (0.98, 1.24) | 1.05 (0.99, 1.10) | 0.50 |
| Black or African American | 2,134 | 222 (10.4) | 1.00 | 1.01 (0.98. 1.06) | 1.05 (0.94, 1.16) | 1.08 (0.91, 1.28) | 1.12 (0.87, 1.44) | 1.05 (0.94, 1.17) |  |
| Hispanic or Latina | 1,124 | 93 (8.3) | 1.00 | 0.99 (0.90, 1.09) | 0.98 (0.79, 1.21) | 0.96 (0.66, 1.39) | 0.94 (0.55, 1.61) | 0.97 (0.77, 1.23) |  |
| BMI (kg/m^2^) |  |  |  |  |  |  |  |  |  |
| <30 | 17,092 | 1,380 (8.1) | 1.00 | 1.01 (0.98, 1.03) | 1.01 (0.96, 1.07) | 1.02 (0.93, 1.12) | 1.03 (0.90, 1.18) | 1.01 (0.95, 1.08) | 0.79 |
| ≥30 | 5,005 | 481 (9.6) | 1.00 | 1.02 (0.99, 1.05) | 1.04 (0.97, 1.12) | 1.07 (0.96, 1.21) | 1.11 (0.94, 1.32) | 1.05 (0.97, 1.13) |  |
| Cancer history |  |  |  |  |  |  |  |  |  |
| No | 19,509 | 1,653 (8.5) | 1.00 | 1.02 (1.00, 1.04) | 1.05 (1.01, 1.10) | 1.09 (1.01, 1.17) | 1.13 (1.01, 1.26) | 1.06 (1.01, 1.11) | 0.10 |
| Yes | 2,588 | 208 (8.0) | 1.00 | 0.97 (0.92, 1.03) | 0.94 (0.83, 1.06) | 0.89 (0.72, 1.10) | 0.85 (0.62, 1.16) | 0.93 (0.81, 1.07) |  |
| Daily MVPA (min/day) |  |  |  |  |  |  |  |  |  |
| <54 | 11,042 | 961 (8.7) | 1.00 | 1.02 (1.00, 1.04) | 1.05 (1.00, 1.10) | 1.09 (1.00, 1.19) | 1.13 (1.00, 1.28) | 1.06 (1.00, 1.12) | 0.67 |
| ≥54 | 11,055 | 900 (8.2) | 1.00 | 1.00 (0.97, 1.04) | 1.01 (0.93, 1.10) | 1.01 (0.88, 1.17) | 1.02 (0.82, 1.26) | 1.01 (0.92, 1.11) |  |
| **13 Physical Activity-Related Cancers** | | | | | | | | | |
| Overall | 20,097 | 1,154 (5.7) | 1.00 | 1.03 (1.01, 1.05) | 1.07 (1.01, 1.12) | 1.12 (1.02, 1.22) | 1.18 (1.04, 1.34) | 1.08 (1.02, 1.14) | - |
| Age (years) |  |  |  |  |  |  |  |  |  |
| <75 | 12,700 | 742 (5.8) | 1.00 | 1.04 (1.01, 1.07) | 1.10 (1.03, 1.17) | 1.17 (1.05, 1.32) | 1.26 (1.07, 1.49) | 1.11 (1.03, 1.19) | 0.16 |
| ≥75 | 7,397 | 412 (5.6) | 1.00 | 1.02 (0.98, 1.05) | 1.04 (0.96, 1.13) | 1.07 (0.93, 1.23) | 1.10 (0.89, 1.35) | 1.04 (0.95, 1.14) |  |
| Race and ethnicity |  |  |  |  |  |  |  |  |  |
| White | 16,743 | 963 (5.8) | 1.00 | 1.03 (1.01, 1.06) | 1.08 (1.02, 1.14) | 1.14 (1.03, 1.25) | 1.20 (1.04, 1.39) | 1.09 (1.02, 1.16) | 0.51 |
| Black or African American | 1,968 | 123 (6.3) | 1.00 | 1.03 (0.97, 1.10) | 1.07 (0.93, 1.23) | 1.12 (0.88, 1.43) | 1.19 (0.83, 1.69) | 1.08 (0.92, 1.26) |  |
| Hispanic or Latina | 1,041 | 51 (4.9) | 1.00 | 0.97 (0.84, 1.10) | 0.92 (0.67, 1.25) | 0.86 (0.51, 1.47) | 0.81 (0.37, 1.75) | 0.91 (0.65, 1.28) |  |
| BMI (kg/m^2^) |  |  |  |  |  |  |  |  |  |
| <30 | 15,553 | 844 (5.4) | 1.00 | 1.01 (0.98, 1.04) | 1.03 (0.96, 1.10) | 1.04 (0.93, 1.18) | 1.07 (0.89, 1.27) | 1.03 (0.95, 1.11) | 0.39 |
| ≥30 | 4,544 | 310 (6.8) | 1.00 | 1.04 (1.00, 1.07) | 1.08 (1.00, 1.18) | 1.15 (0.99, 1.32) | 1.22 (0.99, 1.50) | 1.09 (1.00, 1.20) |  |
| Cancer history |  |  |  |  |  |  |  |  |  |
| No | 19,509 | 1,106 (5.7) | 1.00 | 1.03 (1.01, 1.06) | 1.08 (1.02, 1.13) | 1.13 (1.04, 1.24) | 1.20 (1.05, 1.37) | 1.08 (1.02, 1.15) | 0.049 |
| Yes | 588 | 48 (8.2) | 1.00 | 0.92 (0.80, 1.05) | 0.82 (0.59, 1.12) | 0.71 (0.41, 1.21) | 0.60 (0.27, 1.33) | 0.80 (0.56, 1.13) |  |
| Daily MVPA (min/day) |  |  |  |  |  |  |  |  |  |
| <54 | 9,919 | 587 (5.9) | 1.00 | 1.02 (0.99, 1.05) | 1.05 (0.98, 1.12) | 1.08 (0.97, 1.21) | 1.12 (0.95, 1.63) | 1.05 (0.98, 1.13) | 0.38 |
| ≥54 | 10,178 | 567 (5.6) | 1.00 | 1.04 (0.99, 1.09) | 1.09 (0.98, 1.21) | 1.16 (0.97, 1.40) | 1.25 (0.96, 1.63) | 1.10 (0.98, 1.24) |  |
| **Fatal Cancer** | | | | | | | | | |
| Overall | 22,097 | 601 (2.7) | 1.00 | 1.02 (0.99, 1.05) | 1.04 (0.97, 1.10) | 1.06 (0.95, 1.20) | 1.09 (0.92, 1.30) | 1.04 (0.97, 1.12) | - |
| Age (years) |  |  |  |  |  |  |  |  |  |
| <75 | 13,844 | 241 (1.8) | 1.00 | 0.99 (0.94, 1.05) | 0.99 (0.87, 1.11) | 0.98 (0.79, 1.21) | 0.97 (0.71, 1.31) | 0.99 (0.86, 1.13) | 0.38 |
| ≥75 | 8,253 | 360 (4.4) | 1.00 | 1.04 (1.00, 1.07) | 1.09 (1.00, 1.18) | 1.16 (1.01, 1.32) | 1.23 (1.01, 1.50) | 1.10 (1.01, 1.20) |  |
| Race and ethnicity |  |  |  |  |  |  |  |  |  |
| White | 18,460 | 449 (2.4) | 1.00 | 1.02 (0.99, 1.06) | 1.05 (0.97, 1.14) | 1.08 (0.95, 1.24) | 1.13 (0.92, 1.38) | 1.05 (0.96, 1.15) | 0.76 |
| Black or African American | 2,134 | 99 (4.6) | 1.00 | 1.02 (0.96, 1.08) | 1.04 (0.90, 1.21) | 1.08 (0.84, 1.38) | 1.11 (0.77, 1.60) | 1.05 (0.89, 1.23) |  |
| Hispanic or Latina | 1,124 | 45 (4.0) | 1.00 | 0.98 (0.85, 1.12) | 0.95 (0.69, 1.31) | 0.92 (0.53, 1.58) | 0.88 (0.40, 1.96) | 0.95 (0.66, 1.34) |  |
| BMI (kg/m^2^) |  |  |  |  |  |  |  |  |  |
| <30 | 17,092 | 471 (2.8) | 1.00 | 1.02 (0.98, 1.06) | 1.04 (0.96, 1.14) | 1.08 (0.93, 1.25) | 1.12 (0.90, 1.39) | 1.05 (0.95, 1.16) | 0.92 |
| ≥30 | 5,005 | 130 (2.6) | 1.00 | 1.03 (0.98, 1.08) | 1.07 (0.96, 1.20) | 1.12 (0.93, 1.36) | 1.19 (0.90, 1.57) | 1.08 (0.95, 1.22) |  |
| Cancer history |  |  |  |  |  |  |  |  |  |
| No | 19,509 | 430 (2.2) | 1.00 | 1.00 (0.97, 1.04) | 1.00 (0.92, 1.09) | 1.01 (0.87, 1.16) | 1.01 (0.82, 1.25) | 1.00 (0.92, 1.10) | 0.55 |
| Yes | 2,588 | 171 (6.6) | 1.00 | 1.03 (0.98, 1.08) | 1.07 (0.96, 1.19) | 1.12 (0.93, 1.36) | 1.19 (0.90, 1.56) | 1.08 (0.95, 1.22) |  |
| Daily MVPA (min/day) |  |  |  |  |  |  |  |  |  |
| <54 | 11,042 | 403 (3.6) | 1.00 | 1.01 (0.98, 1.05) | 1.03 (0.96, 1.11) | 1.06 (0.93, 1.20) | 1.09 (0.90, 1.31) | 1.04 (0.95, 1.13) | 0.26 |
| ≥54 | 11,055 | 198 (1.8) | 1.00 | 0.96 (0.89, 1.04) | 0.92 (0.76, 1.11) | 0.86 (0.63, 1.19) | 0.81 (0.50, 1.29) | 0.91 (0.74, 1.12) |  |
| Abbreviations: BMI, body mass index; CHAP, convolutional neural network hip accelerometer posture algorithm; CI, confidence interval; HR, hazard ratio; MVPA, moderate-to-vigorous physical activity; SD, standard deviation; WHAC, Women’s Health Accelerometry Collaboration.  Models were adjusted for age, race and ethnicity, education, smoking status, alcohol use, self-rated general health, postmenopausal hormone use, history of diabetes, and history of cardiovascular disease. Models for each level of race and ethnicity were not mutually adjusted for race and ethnicity.  ^a^ Corresponding percentile values (min/bout): 8.1 (10^th^), 9.9 (25^th^), 12.1 (50^th^), 15.0 (75^th^), 18.2 (90^th^).  ^b^ P-value for multiplicative interaction from likelihood ratio tests | | | | | | | | | |

**Supplemental Table 3.** Associations of CHAP-classified minutes of daily sitting time and mean sitting bout duration with breast cancer incidence (N=20,757)^a^.

|  | **Continuous^c^** | **Quartiles of Sedentary Behavior^b^** | | | |
| --- | --- | --- | --- | --- | --- |
|  |  | **Q1 (Low)** | **Q2** | **Q3** | **Q4 (High)** |
|  | **HR (95% CI)** | **Ref** | **HR (95% CI)** | **HR (95% CI)** | **HR (95% CI)** |
| **WHAC** |  |  |  |  |  |
| Daily sitting time (min/day) |  |  |  |  |  |
| Breast cancer cases [rate]^d^ | 632 [3.6] | 155 [3.5] | 138 [3.2] | 192 [4.6] | 147 [3.7] |
| Model 1^e^ | 1.08 (0.99, 1.17) | 1 (ref) | 0.92 (0.73, 1.16) | 1.33 (1.07, 1.64) | 1.10 (0.87, 1.38) |
| Model 2^e^ | 1.08 (0.99, 1.18) | 1 (ref) | 0.92 (0.73, 1.15) | 1.33 (1.07, 1.65) | 1.10 (0.87, 1.40) |
| Model 2 + MVPA^e^ | 1.07 (0.97, 1.17) | 1 (ref) | 0.91 (0.72, 1.15) | 1.30 (1.04, 1.64) | 1.07 (0.82, 1.39) |
| Model 2 + BMI^e^ | 1.04 (0.95, 1.13) | 1 (ref) | 0.89 (0.71, 1.12) | 1.25 (1.00, 1.55) | 1.00 (0.78, 1.27) |
| Model 2 + physical function^e^ | 1.05 (0.96, 1.14) | 1 (ref) | 0.91 (0.72, 1.15) | 1.30 (1.04, 1.61) | 1.02 (0.80, 1.30) |
| Bout duration (min) |  |  |  |  |  |
| Breast cancer cases [rate]^d^ | 632 [3.6] | 150 [3.5] | 154 [3.6] | 171 [4.0] | 157 [3.8] |
| Model 1^e^ | 1.04 (0.96, 1.13) | 1 (ref) | 1.03 (0.82, 1.29) | 1.16 (0.93, 1.45) | 1.12 (0.89, 1.40) |
| Model 2^e^ | 1.05 (0.96, 1.13) | 1 (ref) | 1.03 (0.82, 1.29) | 1.16 (0.93, 1.44) | 1.12 (0.89, 1.41) |
| Model 2 + MVPA^e^ | 1.02 (0.94, 1.12) | 1 (ref) | 1.02 (0.81, 1.28) | 1.14 (0.90, 1.43) | 1.08 (0.84, 1.39) |
| Model 2 + BMI^e^ | 1.01 (0.93, 1.10) | 1 (ref) | 1.00 (0.80, 1.26) | 1.10 (0.88, 1.38) | 1.03 (0.81, 1.30) |
| Model 2 + physical function^e^ | 1.01 (0.93, 1.10) | 1 (ref) | 1.03 (0.82, 1.29) | 1.10 (0.87, 1.37) | 1.05 (0.83, 1.33) |
| **WHS** |  |  |  |  |  |
| Daily sitting time (min/day) |  |  |  |  |  |
| Breast cancer cases [rate]^d^ | 492 [3.8] | 137 [3.6] | 119 [3.4] | 137 [4.3] | 99 [4.0] |
| Model 1^e^ | 1.07 (0.97, 1.17) | 1 (ref) | 0.95 (0.74, 1.21) | 1.19 (0.94, 1.51) | 1.11 (0.85, 1.44) |
| Model 2^e^ | 1.08 (0.98, 1.19) | 1 (ref) | 0.94 (0.74, 1.21) | 1.21 (0.95, 1.53) | 1.14 (0.88, 1.49) |
| Model 2 + MVPA^e^ | 1.08 (0.96, 1.21) | 1 (ref) | 0.94 (0.73, 1.21) | 1.20 (0.93, 1.55) | 1.14 (0.85, 1.53) |
| Model 2 + BMI^e^ | 1.03 (0.93, 1.14) | 1 (ref) | 0.91 (0.71, 1.17) | 1.12 (0.88, 1.43) | 1.01 (0.77, 1.34) |
| Model 2 + physical function^e^ | 1.05 (0.95, 1.17) | 1 (ref) | 0.94 (0.73, 1.21) | 1.18 (0.92, 1.51) | 1.07 (0.81, 1.42) |
| Bout duration (min) |  |  |  |  |  |
| Breast cancer cases [rate]^d^ | 492 [3.8] | 115 [3.7] | 122 [3.6] | 134 [4.0] | 121 [3.9] |
| Model 1^e^ | 1.02 (0.93, 1.12) | 1 (ref) | 0.96 (0.74, 1.24) | 1.07 (0.83, 1.37) | 1.03 (0.80, 1.33) |
| Model 2^e^ | 1.03 (0.93, 1.13) | 1 (ref) | 0.95 (0.74, 1.23) | 1.07 (0.83, 1.37) | 1.04 (0.81, 1.35) |
| Model 2 + MVPA^e^ | 1.01 (0.91, 1.13) | 1 (ref) | 0.94 (0.73, 1.22) | 1.05 (0.80, 1.36) | 1.01 (0.76, 1.34) |
| Model 2 + BMI^e^ | 0.98 (0.89, 1.09) | 1 (ref) | 0.93 (0.72, 1.20) | 1.01 (0.78, 1.30) | 0.94 (0.72, 1.23) |
| Model 2 + physical function^e^ | 1.00 (0.90, 1.11) | 1 (ref) | 0.96 (0.74, 1.25) | 1.01 (0.78, 1.31) | 0.99 (0.76, 1.29) |
| **OPACH** |  |  |  |  |  |
| Daily sitting time (min/day) |  |  |  |  |  |
| Breast cancer cases [rate]^d^ | 140 [3.2] | 18 [2.8] | 19 [2.3] | 55 [5.4] | 48 [3.2] |
| Model 1^e^ | 1.11 (0.94, 1.32) | 1 (ref) | 0.81 (0.43, 1.55) | 1.97 (1.15, 3.37) | 1.21 (0.69, 2.10) |
| Model 2^e^ | 1.09 (0.92, 1.30) | 1 (ref) | 0.80 (0.42, 1.53) | 1.93 (1.13, 3.31) | 1.15 (0.66, 2.01) |
| Model 2 + MVPA^e^ | 1.05 (0.87, 1.27) | 1 (ref) | 0.78 (0.41, 1.49) | 1.79 (1.03, 3.10) | 1.03 (0.56, 1.87) |
| Model 2 + BMI^e^ | 1.07 (0.90, 1.28) | 1 (ref) | 0.79 (0.41, 1.51) | 1.88 (1.09, 3.23) | 1.09 (0.61, 1.92) |
| Model 2 + physical function^e^ | 1.04 (0.88, 1.24) | 1 (ref) | 0.78 (0.41, 1.49) | 1.85 (1.08, 3.17) | 1.02 (0.57, 1.80) |
| Bout duration (min) |  |  |  |  |  |
| Breast cancer cases [rate]^d^ | 140 [3.2] | 35 [2.8] | 32 [3.5] | 37 [4.1] | 36 [3.7] |
| Model 1^e^ | 1.11 (0.96, 1.27) | 1 (ref) | 1.31 (0.81, 2.12) | 1.50 (0.94, 2.39) | 1.46 (0.91, 2.35) |
| Model 2^e^ | 1.09 (0.95, 1.26) | 1 (ref) | 1.29 (0.80, 2.08) | 1.46 (0.92, 2.33) | 1.40 (0.87, 2.26) |
| Model 2 + MVPA^e^ | 1.06 (0.91, 1.24) | 1 (ref) | 1.28 (0.79, 2.07) | 1.46 (0.91, 2.35) | 1.35 (0.81, 2.24) |
| Model 2 + BMI^e^ | 1.07 (0.93, 1.24) | 1 (ref) | 1.27 (0.78, 2.05) | 1.43 (0.89, 2.29) | 1.34 (0.82, 2.19) |
| Model 2 + physical function^e^ | 1.05 (0.91, 1.22) | 1 (ref) | 1.23 (0.76, 1.99) | 1.38 (0.86, 2.21) | 1.25 (0.76, 2.04) |
| Abbreviations: BMI, body mass index; CHAP, convolutional neural network hip accelerometer posture algorithm; CI, confidence interval; HR, hazard ratio; MVPA, moderate-to-vigorous physical activity; OPACH, Objective Physical Activity and Cardiovascular Health Study; SD, standard deviation; WHAC, Women’s Health Accelerometry Collaboration; WHS, Women’s Health Study.  ^a^ 1,340 women were excluded from this analysis for having prevalent breast cancer at accelerometry baseline.  ^b^ Quartile cut points for sitting time (min/day): <493, 493–567, 568–639, >639. Quartile cut points for bout duration (min): <9.9, 9.9–12.1, 12.2–15.0, >15.0  ^c^ HR and 95% CI per one SD increase in sedentary behavior. For sitting time, SDs were 113 min/day (WHAC), 107 min/day (WHS), and 120 min/day (OPACH). For bout duration, SDs were 4 min (WHAC and WHS) and 5 min (OPACH).  ^d^ Crude incidence rate per 1,000 person-years  ^e^ Model 1 is adjusted for age (years). Model 2 is adjusted for age (years), race and ethnicity (non-Hispanic white, non-Hispanic black, Hispanic, other or unknown), education (high school/GED or less, some college, college graduate), smoking status (current, former, never), alcohol use (never or rarely, monthly, weekly, daily), general health (excellent or very good, good, fair or poor), postmenopausal hormone use (current or otherwise), history of diabetes (yes or no), and history of cardiovascular disease (yes or no). Model 2 + MVPA is adjusted for Model 2 and daily MVPA (min/day).Model 2 + BMI is adjusted for Model 2 and body mass index (<18.5, 18.5-24.9, 25.0-29.9, ≥30 kg/m^2^). Model 2 + physical function is adjusted for Model 2 and physical function (RAND-36 score). | | | | | |

**Supplemental Table 4.** Associations of CHAP-classified minutes of daily sitting time and mean sitting bout duration with endometrial cancer incidence (N=12,770)^a^.

|  | **Continuous^c^** | **Quartiles of Sedentary Behavior^b^** | | | |
| --- | --- | --- | --- | --- | --- |
|  |  | **Q1 (Low)** | **Q2** | **Q3** | **Q4 (High)** |
|  | **HR (95% CI)** | **Ref** | **HR (95% CI)** | **HR (95% CI)** | **HR (95% CI)** |
| **WHAC** |  |  |  |  |  |
| Daily sitting time (min/day) |  |  |  |  |  |
| Endometrial cancer cases [rate]^d^ | 128 [1.2] | 34 [1.2] | 29 [1.0] | 32 [1.2] | 33 [1.4] |
| Model 1^e^ | 1.19 (0.99, 1.44) | 1 (ref) | 0.93 (0.57, 1.53) | 1.12 (0.69, 1.83) | 1.44 (0.88, 2.35) |
| Model 2^e^ | 1.17 (0.96, 1.41) | 1 (ref) | 0.93 (0.57, 1.53) | 1.10 (0.68, 1.80) | 1.37 (0.83, 2.27) |
| Model 2 + MVPA^e^ | 1.13 (0.91, 1.42) | 1 (ref) | 0.90 (0.55, 1.50) | 1.05 (0.63, 1.76) | 1.29 (0.73, 2.26) |
| Model 2 + BMI^e^ | 0.98 (0.80, 1.19) | 1 (ref) | 0.82 (0.50, 1.35) | 0.83 (0.51, 1.37) | 0.88 (0.52, 1.49) |
| Model 2 + physical function^e^ | 1.16 (0.95, 1.42) | 1 (ref) | 0.99 (0.60, 1.64) | 1.13 (0.68, 1.86) | 1.42 (0.85, 2.39) |
| Bout duration (min) |  |  |  |  |  |
| Endometrial cancer cases [rate]^d^ | 128 [1.2] | 29 [1.0] | 26 [0.9] | 33 [1.3] | 40 [1.7] |
| Model 1^e^ | 1.20 (1.02, 1.41) | 1 (ref) | 0.90 (0.53, 1.53) | 1.22 (0.74, 2.01) | 1.67 (1.03, 2.70) |
| Model 2^e^ | 1.17 (0.99, 1.39) | 1 (ref) | 0.89 (0.52, 1.51) | 1.19 (0.72, 1.97) | 1.59 (0.98, 2.60) |
| Model 2 + MVPA^e^ | 1.15 (0.95, 1.39) | 1 (ref) | 0.89 (0.52, 1.52) | 1.19 (0.70, 2.02) | 1.58 (0.91, 2.72) |
| Model 2 + BMI^e^ | 1.03 (0.86, 1.23) | 1 (ref) | 0.81 (0.48, 1.38) | 0.99 (0.59, 1.64) | 1.13 (0.68, 1.88) |
| Model 2 + physical function^e^ | 1.16 (0.98, 1.37) | 1 (ref) | 0.82 (0.48, 1.41) | 1.15 (0.69, 1.91) | 1.57 (0.96, 2.57) |
| **WHS** |  |  |  |  |  |
| Daily sitting time (min/day) |  |  |  |  |  |
| Endometrial cancer cases [rate]^d^ | 108 [1.3] | 30 [1.2] | 24 [1.1] | 30 [1.5] | 24 [1.7] |
| Model 1^e^ | 1.23 (1.00, 1.51) | 1 (ref) | 0.92 (0.54, 1.57) | 1.30 (0.78, 2.16) | 1.49 (0.87, 2.56) |
| Model 2^e^ | 1.20 (0.97, 1.48) | 1 (ref) | 0.91 (0.53, 1.56) | 1.27 (0.76, 2.11) | 1.40 (0.81, 2.44) |
| Model 2 + MVPA^e^ | 1.15 (0.90, 1.46) | 1 (ref) | 0.87 (0.50, 1.50) | 1.17 (0.68, 2.01) | 1.25 (0.67, 2.34) |
| Model 2 + BMI^e^ | 1.00 (0.80, 1.25) | 1 (ref) | 0.81 (0.47, 1.40) | 0.97 (0.57, 1.64) | 0.89 (0.50, 1.60) |
| Model 2 + physical function^e^ | 1.19 (0.96, 1.47) | 1 (ref) | 0.98 (0.57, 1.69) | 1.29 (0.76, 2.18) | 1.43 (0.81, 2.53) |
| Bout duration (min) |  |  |  |  |  |
| Endometrial cancer cases [rate]^d^ | 108 [1.3] | 25 [1.2] | 22 [1.0] | 25 [1.2] | 36 [2.0] |
| Model 1^e^ | 1.25 (1.04, 1.50) | 1 (ref) | 0.83 (0.47, 1.48) | 1.03 (0.59, 1.79) | 1.70 (1.02, 2.84) |
| Model 2^e^ | 1.21 (1.01, 1.46) | 1 (ref) | 0.82 (0.46, 1.46) | 1.00 (0.57, 1.75) | 1.62 (0.96, 2.72) |
| Model 2 + MVPA^e^ | 1.17 (0.94, 1.44) | 1 (ref) | 0.80 (0.45, 1.44) | 0.97 (0.54, 1.74) | 1.51 (0.84, 2.72) |
| Model 2 + BMI^e^ | 1.06 (0.86, 1.29) | 1 (ref) | 0.75 (0.42, 1.34) | 0.83 (0.47, 1.46) | 1.15 (0.67, 1.98) |
| Model 2 + physical function^e^ | 1.19 (0.98, 1.43) | 1 (ref) | 0.75 (0.42, 1.36) | 0.95 (0.54, 1.68) | 1.57 (0.93, 2.65) |
| **OPACH** |  |  |  |  |  |
| Daily sitting time (min/day) |  |  |  |  |  |
| Endometrial cancer cases [rate]^d^ | 20 [0.8] | 4 [1.0] | 5 [1.0] | 2 [0.3] | 9 [1.0] |
| Model 1^e^ | 0.99 (0.64, 1.53) | 1 (ref) | 0.91 (0.24, 3.39) | 0.30 (0.05, 1.64) | 0.92 (0.27, 3.10) |
| Model 2^e^ | 0.98 (0.62, 1.53) | 1 (ref) | 0.95 (0.25, 3.59) | 0.30 (0.05, 1.68) | 0.91 (0.26, 3.16) |
| Model 2 + MVPA^d^ | 1.04 (0.63, 1.72) | 1 (ref) | 1.02 (0.27, 3.87) | 0.35 (0.06, 2.01) | 1.13 (0.29, 4.41) |
| Model 2 + BMI^e^ | 0.88 (0.57, 1.35) | 1 (ref) | 0.72 (0.19, 2.75) | 0.21 (0.04, 1.18) | 0.57 (0.16, 2.07) |
| Model 2 + physical function^e^ | 1.01 (0.64, 1.60) | 1 (ref) | 0.97 (0.26, 3.63) | 0.31 (0.06, 1.75) | 1.01 (0.29, 3.57) |
| Bout duration (min) |  |  |  |  |  |
| Endometrial cancer cases [rate]^d^ | 20 [0.8] | 4 [0.5] | 4 [0.7] | 8 [1.4] | 4 [0.7] |
| Model 1^e^ | 1.02 (0.68, 1.51) | 1 (ref) | 1.24 (0.31, 4.97) | 2.41 (0.72, 8.05) | 1.20 (0.30, 4.86) |
| Model 2^e^ | 1.01 (0.66, 1.53) | 1 (ref) | 1.18 (0.29, 4.77) | 2.40 (0.71, 8.09) | 1.13 (0.27, 4.67) |
| Model 2 + MVPA^d^ | 1.06 (0.69, 1.65) | 1 (ref) | 1.40 (0.34, 5.78) | 3.08 (0.87, 10.93) | 1.57 (0.34, 7.20) |
| Model 2 + BMI^e^ | 0.91 (0.59, 1.41) | 1 (ref) | 1.14 (0.28, 4.62) | 2.02 (0.59, 6.87) | 0.85 (0.20, 3.63) |
| Model 2 + physical function^e^ | 1.04 (0.68, 1.60) | 1 (ref) | 1.23 (0.30, 4.96) | 2.48 (0.74, 8.37) | 1.24 (0.30, 5.20) |
| Abbreviations: BMI, body mass index; CHAP, convolutional neural network hip accelerometer posture algorithm; CI, confidence interval; HR, hazard ratio; MVPA, moderate-to-vigorous physical activity; OPACH, Objective Physical Activity and Cardiovascular Health Study; SD, standard deviation; WHAC, Women’s Health Accelerometry Collaboration; WHS, Women’s Health Study.  ^a^ 9,327 women were excluded from this analysis for having a hysterectomy or prevalent endometrial cancer at accelerometry baseline.  ^b^ Quartile cut points for sitting time (min/day): <493, 493–567, 568–639, >639. Quartile cut points for bout duration (min): <9.9, 9.9–12.1, 12.2–15.0, >15.0  ^c^ HR and 95% CI per one SD increase in sedentary behavior. For sitting time, SDs were 113 min/day (WHAC), 107 min/day (WHS), and 120 min/day (OPACH). For bout duration, SDs were 4 min (WHAC and WHS) and 5 min (OPACH).  ^d^ Crude incidence rate per 1,000 person-years  ^e^ Model 1 is adjusted for age (years). Model 2 is adjusted for age (years), race and ethnicity (non-Hispanic white, non-Hispanic black, Hispanic, other or unknown), education (high school/GED or less, some college, college graduate), smoking status (current, former, never), alcohol use (never or rarely, monthly, weekly, daily), general health (excellent or very good, good, fair or poor), postmenopausal hormone use (current or otherwise), history of diabetes (yes or no), and history of cardiovascular disease (yes or no). Model 2 + MVPA is adjusted for Model 2 and daily MVPA (min/day).Model 2 + BMI is adjusted for Model 2 and body mass index (<18.5, 18.5-24.9, 25.0-29.9, ≥30 kg/m^2^). Model 2 + physical function is adjusted for Model 2 and physical function (RAND-36 score). | | | | | |

**Supplemental Table 5.** Associations of CHAP-classified minutes of daily sitting time and mean sitting bout duration with lung cancer incidence (N=22,004)^a^.

|  | **Continuous^c^** | **Quartiles of Sedentary Behavior^b^** | | | |
| --- | --- | --- | --- | --- | --- |
|  |  | **Q1 (Low)** | **Q2** | **Q3** | **Q4 (High)** |
|  | **HR (95% CI)** | **Ref** | **HR (95% CI)** | **HR (95% CI)** | **HR (95% CI)** |
| **WHAC** |  |  |  |  |  |
| Daily sitting time (min/day) |  |  |  |  |  |
| Lung cancer cases [rate]^d^ | 207 [1.1] | 38 [0.8] | 42 [0.9] | 64 [1.4] | 63 [1.5] |
| Model 1^e^ | 1.17 (1.01, 1.35) | 1 (ref) | 1.07 (0.69, 1.66) | 1.58 (1.06, 2.38) | 1.47 (0.97, 2.23) |
| Model 2^e^ | 1.06 (0.92, 1.23) | 1 (ref) | 1.03 (0.66, 1.60) | 1.42 (0.95, 2.13) | 1.18 (0.77, 1.80) |
| Model 2 + MVPA^e^ | 1.10 (0.94, 1.31) | 1 (ref) | 1.08 (0.69, 1.69) | 1.54 (1.00, 2.36) | 1.31 (0.82, 2.10) |
| Model 2 + BMI^e^ | 1.08 (0.93, 1.26) | 1 (ref) | 1.03 (0.67, 1.61) | 1.45 (0.96, 2.19) | 1.24 (0.80, 1.92) |
| Model 2 + physical function^e^ | 1.04 (0.89, 1.21) | 1 (ref) | 1.10 (0.70, 1.72) | 1.39 (0.91, 2.13) | 1.11 (0.71, 1.74) |
| Bout duration (min) |  |  |  |  |  |
| Lung cancer cases [rate]^d^ | 207 [1.1] | 56 [1.2] | 53 [1.1] | 45 [1.0] | 53 [1.2] |
| Model 1^e^ | 1.06 (0.94, 1.21) | 1 (ref) | 0.96 (0.66, 1.40) | 0.81 (0.55, 1.21) | 0.95 (0.65, 1.39) |
| Model 2^e^ | 1.04 (0.91, 1.18) | 1 (ref) | 0.98 (0.67, 1.43) | 0.79 (0.53, 1.18) | 0.90 (0.62, 1.33) |
| Model 2 + MVPA^e^ | 1.06 (0.92, 1.22) | 1 (ref) | 0.98 (0.67, 1.44) | 0.80 (0.53, 1.20) | 0.91 (0.60, 1.38) |
| Model 2 + BMI^e^ | 1.05 (0.92, 1.21) | 1 (ref) | 0.99 (0.68, 1.44) | 0.80 (0.54, 1.19) | 0.93 (0.62, 1.38) |
| Model 2 + physical function^e^ | 1.04 (0.91, 1.19) | 1 (ref) | 1.01 (0.69, 1.50) | 0.79 (0.52, 1.19) | 0.87 (0.58, 1.30) |
| **WHS** |  |  |  |  |  |
| Daily sitting time (min/day) |  |  |  |  |  |
| Lung cancer cases [rate]^d^ | 131 [0.9] | 26 [0.6] | 29 [0.8] | 44 [1.3] | 32 [1.2] |
| Model 1^e^ | 1.32 (1.10, 1.60) | 1 (ref) | 1.18 (0.70, 2.01) | 1.95 (1.20, 3.17) | 1.81 (1.07, 3.04) |
| Model 2^e^ | 1.14 (0.94, 1.37) | 1 (ref) | 1.11 (0.65, 1.89) | 1.65 (1.01, 2.70) | 1.27 (0.74, 2.17) |
| Model 2 + MVPA^e^ | 1.22 (0.97, 1.52) | 1 (ref) | 1.19 (0.69, 2.04) | 1.82 (1.08, 3.06) | 1.47 (0.80, 2.68) |
| Model 2 + BMI^e^ | 1.16 (0.95, 1.42) | 1 (ref) | 1.10 (0.65, 1.87) | 1.66 (1.01, 2.72) | 1.34 (0.77, 2.34) |
| Model 2 + physical function^e^ | 1.10 (0.90, 1.35) | 1 (ref) | 1.22 (0.71, 2.12) | 1.61 (0.96, 2.72) | 1.17 (0.66, 2.10) |
| Bout duration (min) |  |  |  |  |  |
| Lung cancer cases [rate]^d^ | 131 [0.9] | 28 [0.9] | 37 [1.0] | 30 [0.8] | 36 [1.1] |
| Model 1^e^ | 1.09 (0.92, 1.30) | 1 (ref) | 1.16 (0.71, 1.90) | 0.95 (0.56, 1.59) | 1.20 (0.73, 1.98) |
| Model 2^e^ | 1.04 (0.87, 1.24) | 1 (ref) | 1.21 (0.74, 1.97) | 0.92 (0.55, 1.55) | 1.11 (0.67, 1.84) |
| Model 2 + MVPA^e^ | 1.07 (0.88, 1.30) | 1 (ref) | 1.22 (0.74, 2.02) | 0.95 (0.55, 1.64) | 1.16 (0.66, 2.04) |
| Model 2 + BMI^e^ | 1.06 (0.88, 1.27) | 1 (ref) | 1.20 (0.73, 1.96) | 0.91 (0.54, 1.54) | 1.13 (0.68, 1.90) |
| Model 2 + physical function^e^ | 1.04 (0.87, 1.26) | 1 (ref) | 1.32 (0.79, 2.23) | 0.95 (0.55, 1.65) | 1.12 (0.66, 1.93) |
| **OPACH** |  |  |  |  |  |
| Daily sitting time (min/day) |  |  |  |  |  |
| Lung cancer cases [rate]^d^ | 76 [1.6] | 12 [1.8] | 13 [1.5] | 20 [1.8] | 31 [1.9] |
| Model 1^e^ | 0.97 (0.78, 1.21) | 1 (ref) | 0.80 (0.36, 1.76) | 0.94 (0.46, 1.94) | 0.93 (0.47, 1.84) |
| Model 2^e^ | 0.95 (0.76, 1.18) | 1 (ref) | 0.86 (0.39, 1.90) | 0.93 (0.45, 1.91) | 0.89 (0.45, 1.77) |
| Model 2 + MVPA^e^ | 0.96 (0.76, 1.23) | 1 (ref) | 0.88 (0.40, 1.95) | 0.99 (0.47, 2.07) | 0.96 (0.46, 2.03) |
| Model 2 + BMI^e^ | 0.97 (0.77, 1.21) | 1 (ref) | 0.90 (0.41, 1.99) | 0.99 (0.48, 2.06) | 0.96 (0.48, 1.95) |
| Model 2 + physical function^e^ | 0.94 (0.76, 1.18) | 1 (ref) | 0.87 (0.39, 1.91) | 0.94 (0.46, 1.94) | 0.89 (0.44, 1.79) |
| Bout duration (min) |  |  |  |  |  |
| Lung cancer cases [rate]^d^ | 76 [1.6] | 28 [2.1] | 16 [1.7] | 15 [1.6] | 17 [1.7] |
| Model 1^e^ | 1.03 (0.85, 1.26) | 1 (ref) | 0.73 (0.40, 1.36) | 0.68 (0.36, 1.28) | 0.67 (0.36, 1.24) |
| Model 2^e^ | 1.04 (0.85, 1.26) | 1 (ref) | 0.74 (0.40, 1.38) | 0.68 (0.36, 1.28) | 0.67 (0.36, 1.24) |
| Model 2 + MVPA^e^ | 1.06 (0.86, 1.31) | 1 (ref) | 0.75 (0.40, 1.39) | 0.67 (0.35, 1.29) | 0.66 (0.34, 1.27) |
| Model 2 + BMI^e^ | 1.06 (0.86, 1.30) | 1 (ref) | 0.76 (0.41, 1.41) | 0.70 (0.37, 1.32) | 0.69 (0.36, 1.31) |
| Model 2 + physical function^e^ | 1.04 (0.85, 1.28) | 1 (ref) | 0.74 (0.40, 1.37) | 0.69 (0.36, 1.30) | 0.63 (0.33, 1.20) |
| Abbreviations: BMI, body mass index; CHAP, convolutional neural network hip accelerometer posture algorithm; CI, confidence interval; HR, hazard ratio; MVPA, moderate-to-vigorous physical activity; OPACH, Objective Physical Activity and Cardiovascular Health Study; SD, standard deviation; WHAC, Women’s Health Accelerometry Collaboration; WHS, Women’s Health Study.  ^a^ 93 women were excluded from this analysis for having prevalent lung cancer at accelerometry baseline.  ^b^ Quartile cut points for sitting time (min/day): <493, 493–567, 568–639, >639. Quartile cut points for bout duration (min): <9.9, 9.9–12.1, 12.2–15.0, >15.0  ^c^ HR and 95% CI per one SD increase in sedentary behavior. For sitting time, SDs were 113 min/day (WHAC), 107 min/day (WHS), and 120 min/day (OPACH). For bout duration, SDs were 4 min (WHAC and WHS) and 5 min (OPACH).  ^d^ Crude incidence rate per 1,000 person-years  ^e^ Model 1 is adjusted for age (years). Model 2 is adjusted for age (years), race and ethnicity (non-Hispanic white, non-Hispanic black, Hispanic, other or unknown), education (high school/GED or less, some college, college graduate), smoking status (current, former, never), alcohol use (never or rarely, monthly, weekly, daily), general health (excellent or very good, good, fair or poor), postmenopausal hormone use (current or otherwise), history of diabetes (yes or no), and history of cardiovascular disease (yes or no). Model 2 + MVPA is adjusted for Model 2 and daily MVPA (min/day).Model 2 + BMI is adjusted for Model 2 and body mass index (<18.5, 18.5-24.9, 25.0-29.9, ≥30 kg/m^2^). Model 2 + physical function is adjusted for Model 2 and physical function (RAND-36 score). | | | | | |

**Supplemental Table 6.** Associations of CHAP-classified minutes of daily sitting time and mean sitting bout duration with colon cancer incidence (N=21,919)^a^.

|  | **Continuous^c^** | **Quartiles of Sedentary Behavior^b^** | | | |
| --- | --- | --- | --- | --- | --- |
|  |  | **Q1 (Low)** | **Q2** | **Q3** | **Q4 (High)** |
|  | **HR (95% CI)** | **Ref** | **HR (95% CI)** | **HR (95% CI)** | **HR (95% CI)** |
| **WHAC** |  |  |  |  |  |
| Daily sitting time (min/day) |  |  |  |  |  |
| Colon cancer cases [rate]^d^ | 116 [0.6] | 23 [0.5] | 30 [0.6] | 29 [0.6] | 34 [0.8] |
| Model 1^e^ | 1.00 (0.83, 1.21) | 1 (ref) | 1.19 (0.69, 2.05) | 1.07 (0.62, 1.86) | 1.08 (0.63, 1.88) |
| Model 2^e^ | 1.02 (0.84, 1.25) | 1 (ref) | 1.20 (0.70, 2.07) | 1.09 (0.62, 1.89) | 1.16 (0.66, 2.03) |
| Model 2 + MVPA^e^ | 1.01 (0.81, 1.26) | 1 (ref) | 1.20 (0.69, 2.08) | 1.07 (0.60, 1.90) | 1.11 (0.60, 2.04) |
| Model 2 + BMI^e^ | 0.97 (0.79, 1.19) | 1 (ref) | 1.17 (0.67, 2.02) | 1.01 (0.57, 1.78) | 1.02 (0.57, 1.82) |
| Model 2 + physical function^e^ | 1.06 (0.86, 1.30) | 1 (ref) | 1.27 (0.72, 2.23) | 1.12 (0.63, 2.00) | 1.26 (0.70, 2.27) |
| Bout duration (min) |  |  |  |  |  |
| Colon cancer cases [rate]^d^ | 116 [0.6] | 30 [0.7] | 31 [0.7] | 17 [0.4] | 38 [0.9] |
| Model 1^e^ | 1.02 (0.86, 1.22) | 1 (ref) | 0.99 (0.60, 1.64) | 0.53 (0.29, 0.95) | 1.10 (0.68, 1.78) |
| Model 2^e^ | 1.04 (0.87, 1.24) | 1 (ref) | 0.99 (0.60, 1.65) | 0.53 (0.29, 0.97) | 1.14 (0.70, 1.85) |
| Model 2 + MVPA^e^ | 1.03 (0.85, 1.25) | 1 (ref) | 0.97 (0.58, 1.62) | 0.52 (0.28, 0.95) | 1.07 (0.63, 1.83) |
| Model 2 + BMI^e^ | 0.99 (0.82, 1.19) | 1 (ref) | 0.97 (0.59, 1.61) | 0.50 (0.28, 0.92) | 1.02 (0.61, 1.69) |
| Model 2 + physical function^e^ | 1.03 (0.85, 1.24) | 1 (ref) | 1.07 (0.64, 1.79) | 0.58 (0.31, 1.06) | 1.14 (0.68, 1.90) |
| **WHS** |  |  |  |  |  |
| Daily sitting time (min/day) |  |  |  |  |  |
| Colon cancer cases [rate]^d^ | 73 [0.5] | 18 [0.4] | 21 [0.6] | 23 [0.7] | 11 [0.4] |
| Model 1^e^ | 0.90 (0.71, 1.15) | 1 (ref) | 1.17 (0.62, 2.19) | 1.35 (0.73, 2.50) | 0.79 (0.37, 1.68) |
| Model 2^e^ | 0.90 (0.70, 1.15) | 1 (ref) | 1.17 (0.62, 2.20) | 1.33 (0.72, 2.49) | 0.77 (0.36, 1.67) |
| Model 2 + MVPA^e^ | 0.88 (0.66, 1.16) | 1 (ref) | 1.17 (0.61, 2.22) | 1.34 (0.69, 2.58) | 0.78 (0.34, 1.79) |
| Model 2 + BMI^e^ | 0.82 (0.63, 1.06) | 1 (ref) | 1.13 (0.60, 2.13) | 1.19 (0.63, 2.26) | 0.62 (0.28, 1.38) |
| Model 2 + physical function^e^ | 0.89 (0.69, 1.16) | 1 (ref) | 1.23 (0.64, 2.39) | 1.33 (0.69, 2.58) | 0.78 (0.35, 1.75) |
| Bout duration (min) |  |  |  |  |  |
| Colon cancer cases [rate]^d^ | 73 [0.5] | 17 [0.5] | 22 [0.6] | 10 [0.3] | 24 [0.7] |
| Model 1^e^ | 1.03 (0.81, 1.31) | 1 (ref) | 1.11 (0.59, 2.09) | 0.48 (0.22, 1.05) | 1.19 (0.64, 2.23) |
| Model 2^e^ | 1.03 (0.82, 1.31) | 1 (ref) | 1.12 (0.59, 2.12) | 0.48 (0.22, 1.05) | 1.19 (0.63, 2.24) |
| Model 2 + MVPA^e^ | 1.05 (0.80, 1.37) | 1 (ref) | 1.13 (0.59, 2.15) | 0.49 (0.22, 1.10) | 1.23 (0.61, 2.49) |
| Model 2 + BMI^e^ | 0.97 (0.75, 1.25) | 1 (ref) | 1.10 (0.58, 2.07) | 0.44 (0.20, 0.98) | 1.03 (0.53, 2.00) |
| Model 2 + physical function^e^ | 0.97 (0.75, 1.26) | 1 (ref) | 1.28 (0.66, 2.47) | 0.53 (0.24, 1.20) | 1.12 (0.57, 2.22) |
| **OPACH** |  |  |  |  |  |
| Daily sitting time (min/day) |  |  |  |  |  |
| Colon cancer cases [rate]^d^ | 43 [0.9] | 5 [0.7] | 9 [1.0] | 6 [0.6] | 23 [1.4] |
| Model 1^e^ | 1.18 (0.86, 1.62) | 1 (ref) | 1.23 (0.41, 3.68) | 0.59 (0.18, 1.95) | 1.30 (0.49, 3.47) |
| Model 2^e^ | 1.30 (0.94, 1.81) | 1 (ref) | 1.25 (0.42, 3.75) | 0.64 (0.19, 2.10) | 1.58 (0.59, 4.24) |
| Model 2 + MVPA^e^ | 1.25 (0.87, 1.80) | 1 (ref) | 1.26 (0.42, 3.78) | 0.62 (0.18, 2.06) | 1.42 (0.50, 4.04) |
| Model 2 + BMI^e^ | 1.28 (0.91, 1.80) | 1 (ref) | 1.22 (0.41, 3.67) | 0.61 (0.18, 2.04) | 1.48 (0.54, 4.08) |
| Model 2 + physical function^e^ | 1.42 (1.01, 2.01) | 1 (ref) | 1.31 (0.44, 3.92) | 0.70 (0.21, 2.30) | 1.90 (0.70, 5.16) |
| Bout duration (min) |  |  |  |  |  |
| Colon cancer cases [rate]^d^ | 43 [0.9] | 13 [1.0] | 9 [0.9] | 7 [0.7] | 14 [1.4] |
| Model 1^e^ | 1.03 (0.80, 1.32) | 1 (ref) | 0.82 (0.35, 1.92) | 0.63 (0.25, 1.57) | 0.99 (0.46, 2.11) |
| Model 2^e^ | 1.08 (0.84, 1.38) | 1 (ref) | 0.86 (0.37, 2.01) | 0.67 (0.27, 1.69) | 1.13 (0.52, 2.44) |
| Model 2 + MVPA^e^ | 1.02 (0.78, 1.34) | 1 (ref) | 0.79 (0.33, 1.86) | 0.60 (0.23, 1.53) | 0.93 (0.40, 2.13) |
| Model 2 + BMI^e^ | 1.06 (0.81, 1.37) | 1 (ref) | 0.83 (0.35, 1.96) | 0.65 (0.26, 1.64) | 1.05 (0.47, 2.33) |
| Model 2 + physical function^e^ | 1.14 (0.89, 1.45) | 1 (ref) | 0.88 (0.38, 2.07) | 0.71 (0.28, 1.80) | 1.33 (0.60, 2.92) |
| Abbreviations: BMI, body mass index; CHAP, convolutional neural network hip accelerometer posture algorithm; CI, confidence interval; HR, hazard ratio; MVPA, moderate-to-vigorous physical activity; OPACH, Objective Physical Activity and Cardiovascular Health Study; SD, standard deviation; WHAC, Women’s Health Accelerometry Collaboration; WHS, Women’s Health Study.  ^a^ 178 women were excluded from this analysis for having prevalent colon cancer at accelerometry baseline.  ^b^ Quartile cut points for sitting time (min/day): <493, 493–567, 568–639, >639. Quartile cut points for bout duration (min): <9.9, 9.9–12.1, 12.2–15.0, >15.0  ^c^ HR and 95% CI per one SD increase in sedentary behavior. For sitting time, SDs were 113 min/day (WHAC), 107 min/day (WHS), and 120 min/day (OPACH). For bout duration, SDs were 4 min (WHAC and WHS) and 5 min (OPACH).  ^d^ Crude incidence rate per 1,000 person-years  ^e^ Model 1 is adjusted for age (years). Model 2 is adjusted for age (years), race and ethnicity (non-Hispanic white, non-Hispanic black, Hispanic, other or unknown), education (high school/GED or less, some college, college graduate), smoking status (current, former, never), alcohol use (never or rarely, monthly, weekly, daily), general health (excellent or very good, good, fair or poor), postmenopausal hormone use (current or otherwise), history of diabetes (yes or no), and history of cardiovascular disease (yes or no). Model 2 + MVPA is adjusted for Model 2 and daily MVPA (min/day).Model 2 + BMI is adjusted for Model 2 and body mass index (<18.5, 18.5-24.9, 25.0-29.9, ≥30 kg/m^2^). Model 2 + physical function is adjusted for Model 2 and physical function (RAND-36 score). | | | | | |

**Supplemental Table 7.** Associations of CHAP-classified minutes of daily sitting time and mean sitting bout duration with fatal cancer after excluding deaths within the first 2 years of follow-up (n=70 deaths excluded)

|  | **Continuous^b^** | **Quartiles of Sedentary Behavior^a^** | | | |
| --- | --- | --- | --- | --- | --- |
|  |  | **Q1 (Low)** | **Q2** | **Q3** | **Q4 (High)** |
|  | **HR (95% CI)** | **Ref** | **HR (95% CI)** | **HR (95% CI)** | **HR (95% CI)** |
| **WHAC** |  |  |  |  |  |
| Daily sitting time (min/day) |  |  |  |  |  |
| Cancer cases [rate]^c^ | 531 [2.9] | 107 [2.2] | 120 [2.5] | 117 [2.5] | 187 [4.3] |
| Model 1^d^ | 1.10 (1.01, 1.21) | 1 (ref) | 1.03 (0.79, 1.33) | 0.93 (0.71, 1.21) | 1.27 (0.99, 1.62) |
| Model 2^d^ | 1.05 (0.96, 1.15) | 1 (ref) | 1.01 (0.77, 1.31) | 0.87 (0.67, 1.14) | 1.13 (0.88, 1.46) |
| Model 2 + MVPA^d^ | 1.02 (0.92, 1.12) | 1 (ref) | 0.97 (0.75, 1.27) | 0.82 (0.62, 1.08) | 1.03 (0.78, 1.35) |
| Model 2 + BMI^d^ | 1.07 (0.97, 1.17) | 1 (ref) | 1.01 (0.78, 1.32) | 0.89 (0.68, 1.17) | 1.17 (0.90, 1.53) |
| Model 2 + physical function^d^ | 1.05 (0.95, 1.15) | 1 (ref) | 0.98 (0.74, 1.29) | 0.85 (0.64, 1.13) | 1.11 (0.84, 1.45) |
| Bout duration (min) |  |  |  |  |  |
| Cancer cases [rate]^c^ | 531 [2.9] | 135 [2.9] | 130 [2.7] | 115 [2.5] | 151 [3.4] |
| Model 1^d^ | 1.05 (0.97, 1.14) | 1 (ref) | 1.00 (0.78, 1.27) | 0.88 (0.68, 1.12) | 1.10 (0.87, 1.39) |
| Model 2^d^ | 1.03 (0.95, 1.12) | 1 (ref) | 0.99 (0.78, 1.26) | 0.85 (0.66, 1.10) | 1.05 (0.82, 1.33) |
| Model 2 + MVPA^d^ | 1.00 (0.92, 1.09) | 1 (ref) | 0.96 (0.75, 1.22) | 0.80 (0.62, 1.04) | 0.94 (0.73, 1.22) |
| Model 2 + BMI^d^ | 1.04 (0.96, 1.13) | 1 (ref) | 1.00 (0.78, 1.27) | 0.87 (0.67, 1.12) | 1.07 (0.84, 1.37) |
| Model 2 + physical function^d^ | 1.02 (0.93, 1.11) | 1 (ref) | 1.02 (0.79, 1.31) | 0.87 (0.67, 1.14) | 1.02 (0.79, 1.32) |
| **WHS** |  |  |  |  |  |
| Daily sitting time (min/day) |  |  |  |  |  |
| Cancer cases [rate]^c^ | 276 [1.9] | 77 [1.9] | 78 [2.0] | 63 [1.8] | 58 [2.1] |
| Model 1^d^ | 1.01 (0.89, 1.15) | 1 (ref) | 1.04 (0.76, 1.43) | 0.89 (0.64, 1.25) | 1.05 (0.74, 1.48) |
| Model 2^d^ | 0.93 (0.82, 1.06) | 1 (ref) | 1.00 (0.73, 1.37) | 0.82 (0.58, 1.14) | 0.87 (0.61, 1.24) |
| Model 2 + MVPA^d^ | 0.89 (0.78, 1.02) | 1 (ref) | 0.91 (0.65, 1.28) | 0.86 (0.61, 1.22) | 0.73 (0.50, 1.07) |
| Model 2 + BMI^d^ | 0.95 (0.83, 1.09) | 1 (ref) | 1.00 (0.73, 1.38) | 0.83 (0.59, 1.17) | 0.91 (0.63, 1.32) |
| Model 2 + physical function^d^ | 0.92 (0.80, 1.06) | 1 (ref) | 0.97 (0.69, 1.37) | 0.77 (0.53, 1.12) | 0.83 (0.56, 1.22) |
| Bout duration (min) |  |  |  |  |  |
| Cancer cases [rate]^c^ | 276 [1.9] | 65 [1.9] | 80 [2.1] | 57 [1.6] | 74 [2.1] |
| Model 1^d^ | 0.98 (0.86, 1.11) | 1 (ref) | 1.06 (0.76, 1.46) | 0.74 (0.52, 1.06) | 1.02 (0.73, 1.43) |
| Model 2^d^ | 0.94 (0.83, 1.08) | 1 (ref) | 1.05 (0.76, 1.46) | 0.72 (0.50, 1.03) | 0.95 (0.68, 1.34) |
| Model 2 + MVPA^d^ | 0.91 (0.79, 1.04) | 1 (ref) | 0.99 (0.71, 1.38) | 0.67 (0.47, 0.97) | 0.84 (0.58, 1.21) |
| Model 2 + BMI^d^ | 0.96 (0.84, 1.10) | 1 (ref) | 1.06 (0.76, 1.48) | 0.73 (0.51, 1.05) | 0.99 (0.70, 1.41) |
| Model 2 + physical function^d^ | 0.93 (0.81, 1.08) | 1 (ref) | 1.17 (0.81, 1.68) | 0.76 (0.51, 1.13) | 0.98 (0.67, 1.43) |
| **OPACH** |  |  |  |  |  |
| Daily sitting time (min/day) |  |  |  |  |  |
| Cancer cases [rate]^c^ | 255 [6.0] | 30 [4.3] | 42 [4.6] | 54 [4.8] | 129 [7.8] |
| Model 1^d^ | 1.21 (1.06, 1.38) | 1 (ref) | 1.03 (0.65, 1.65) | 1.02 (0.65, 1.60) | 1.53 (1.02, 2.30) |
| Model 2^d^ | 1.18 (1.03, 1.35) | 1 (ref) | 1.04 (0.65, 1.67) | 1.01 (0.64, 1.58) | 1.46 (0.97, 2.20) |
| Model 2 + MVPA^d^ | 1.16 (1.00, 1.35) | 1 (ref) | 1.01 (0.68, 1.51) | 1.01 (0.70, 1.49) | 1.43 (0.95, 2.15) |
| Model 2 + BMI^d^ | 1.19 (1.04, 1.37) | 1 (ref) | 1.05 (0.66, 1.68) | 1.03 (0.66, 1.62) | 1.51 (0.99, 2.29) |
| Model 2 + physical function^d^ | 1.17 (1.02, 1.34) | 1 (ref) | 1.01 (0.63, 1.62) | 1.01 (0.64, 1.58) | 1.43 (0.94, 2.17) |
| Bout duration (min) |  |  |  |  |  |
| Cancer cases [rate]^c^ | 255 [6.0] | 70 [5.2] | 50 [5.0] | 58 [5.9] | 77 [7.3] |
| Model 1^d^ | 1.10 (1.00, 1.21) | 1 (ref) | 0.90 (0.63, 1.30) | 1.05 (0.74, 1.49) | 1.18 (0.85, 1.64) |
| Model 2^d^ | 1.09 (0.98, 1.20) | 1 (ref) | 0.89 (0.62, 1.28) | 1.02 (0.72, 1.45) | 1.13 (0.81, 1.57) |
| Model 2 + MVPA^d^ | 1.07 (0.95, 1.22) | 1 (ref) | 0.97 (0.67, 1.40) | 0.99 (0.68, 1.43) | 1.06 (0.73, 1.55) |
| Model 2 + BMI^d^ | 1.10 (0.99, 1.21) | 1 (ref) | 0.89 (0.62, 1.29) | 1.03 (0.73, 1.47) | 1.14 (0.81, 1.61) |
| Model 2 + physical function^d^ | 1.07 (0.97, 1.19) | 1 (ref) | 0.86 (0.60, 1.24) | 1.00 (0.70, 1.43) | 1.07 (0.76, 1.50) |
| Abbreviations: BMI, body mass index; CHAP, convolutional neural network hip accelerometer posture algorithm; CI, confidence interval; HR, hazard ratio; MVPA, moderate-to-vigorous physical activity; OPACH, Objective Physical Activity and Cardiovascular Health Study; SD, standard deviation; WHAC, Women’s Health Accelerometry Collaboration; WHS, Women’s Health Study.  ^a^ Adjusted for awake wear time using the residuals method. Quartile cut points for sitting time (min/day): <493, 493–567, 568–639, >639. Quartile cut points for bout duration (min): <9.9, 9.9–12.1, 12.2–15.0, >15.0  ^b^ HR and 95% CI per one SD increase in sedentary behavior. For sitting time, SDs were 113 min/day (WHAC), 107 min/day (WHS), and 120 min/day (OPACH). For bout duration, SDs were 4 min (WHAC and WHS) and 5 min (OPACH).  ^c^ Crude incidence rate per 1,000 person-years  ^d^ Model 1 is adjusted for age (years). Model 2 is adjusted for age (years), race and ethnicity (non-Hispanic white, non-Hispanic black, Hispanic, other or unknown), education (high school/GED or less, some college, college graduate), smoking status (current, former, never), alcohol use (never or rarely, monthly, weekly, daily), general health (excellent or very good, good, fair or poor), postmenopausal hormone use (current or otherwise), history of diabetes (yes or no), and history of cardiovascular disease (yes or no). Model 2 + MVPA is adjusted for Model 2 and daily MVPA (min/day).Model 2 + BMI is adjusted for Model 2 and body mass index (<18.5, 18.5-24.9, 25.0-29.9, ≥30 kg/m^2^). Model 2 + physical function is adjusted for Model 2 and physical function (RAND-36 score). | | | | | |
